# Northern Latitude but Not Season Is Associated with Increased Rates of Hospitalizations Related to Inflammatory Bowel Disease: Results of a Multi-Year Analysis of a National Cohort

**DOI:** 10.1371/journal.pone.0161523

**Published:** 2016-08-31

**Authors:** Adam C. Stein, John Nick Gaetano, Jeffrey Jacobs, Rangesh Kunnavakkam, Marc Bissonnette, Joel Pekow

**Affiliations:** 1 Department of Medicine, Section of Gastroenterology, Hepatology, and Nutrition, University of Chicago, Chicago, Illinois, United States of America; 2 Department of Health Studies, University of Chicago, Chicago, Illinois, United States of America; National Cancer Institute, UNITED STATES

## Abstract

**Background and Aims:**

There is growing evidence that the incidence and severity of inflammatory bowel disease (IBD) may be geographically and seasonally related. Why these associations are observed remains unclear. We assessed the impact of geographic location, season, and exposure to ultraviolet light on disease severity by measuring national hospital IBD-related discharge rates.

**Methods:**

Utilizing the Nationwide Inpatient Sample (NIS), we identified all patients with IBD-related discharges from 2001–2007. Patients were included if they were discharged from states above the 40^th^ parallel (north) or at or below the 35^th^ parallel (south); and their discharge fell within the winter (January, February, and March) or summer (July, August, and September). Groups of patients were assessed comparing north to south within each season, and summer to winter within each region. UV index was recorded from the National Weather Service data and compared to monthly discharge rates.

**Results:**

There was a consistent pattern of increased IBD-related hospitalization rates in northern states compared to southern states for both ulcerative colitis and Crohn’s disease. Differences in IBD-related hospitalization rates by season, however, were not uniform across the years studied. UV index was significantly inversely associated although not proportional to discharge rates for both Crohn’s disease and ulcerative colitis.

**Conclusions:**

In the US, there is a significant increased rate of IBD-related hospitalizations in the northern compared to southern states, which not fully explained by differences in UV exposure.

## Introduction

Inflammatory bowel diseases (IBD), including Crohn’s disease (CD) and ulcerative colitis (UC), are a spectrum of chronic inflammatory diseases of unknown etiology. It is hypothesized that IBD arises in genetically susceptible individuals who have environmental exposure(s) leading to aberrant immune responses. While numerous environmental factors have been postulated, there remains much controversy as to the causal roles of these potential disease triggers. [1]

One compelling and consistent finding is the observed increase in incidence of both CD and UC in northern latitudes compared to southern latitudes in the northern hemisphere. [1,2] [3–7] Given the multitude of both measurable and unknown variables potentially associated with this latitudinal gradient, it remains difficult to associate specific factors with causality. Nonetheless, numerous hypotheses exist to explain the differences in incidence over latitudinal gradient, including differences in the geographic environment, socioeconomic factors, temperature variation, and sunlight exposure.

Recently published evidence from cohorts in France shows that higher sun exposure by ultraviolet (UV) dose is associated with decreased incidence of CD, whereas no association was found for UC. [8] Other studies from the United States concluded that increased UV light exposure decreases both the risk of hospital admission for patients with IBD and the likelihood of inpatient surgery in CD. [9,10] One potential mechanistic explanation for these findings relates to differences in serum vitamin D levels. Sunlight exposure in northern latitudes is limited both by oblique incidence of the sun rays as well as colder temperatures limiting skin exposure to UV light. Solar UVB converts 7-dehydroxycholesterol in the skin to previtamin D3 that in turn is metabolized to vitamin D3 [25(OH)D]. 25(OH)D is metabolized into 1,25 dehydroxyvitamin D (1,25(OH)_2_D_3_)_,_ which has been shown to suppress inflammation in vitro and in animal studies. [11–13] Consistent with these preclinical studies, higher serum vitamin D levels are associated with a decreased risk of IBD as well as a decrease in symptom severity. [14,15]

While vitamin D levels are inversely associated with disease activity in IBD, it remains unclear if there is an association between disease activity and the north-south latitudinal gradient. [4,16] Furthermore, findings of studies evaluating the impact of seasonal variation on disease incidence as well as activity are inconsistent despite the fact that vitamin D levels are known to fluctuate by season. [17,18] [16,19–22] This suggests that there may be a role with variation in long-term sunlight exposure affecting IBD onset, but the short-term impact of seasonal changes in sunlight exposure on symptoms remains unclear. Many of the aforementioned studies, however, have not controlled for geography in their investigations into the role of seasonal variation on IBD symptoms. Sunlight exposure has tremendous seasonal variation significantly with increasing latitude. As such, comparing two regions with different latitudes while controlling for seasonal variability allows for closer study of long term sunlight exposure, as well as other environmental differences between northern and southern regions, to assess changes in disease activity.

In this study we sought to assess the impact of latitude, season, and UV exposure on IBD disease activity in the continental United States using UV index forecast from the National Weather Service Climate Prediction Center and the largest all-payer national database measuring hospital discharges, the Nationwide Inpatient Sample (NIS).

## Materials and Methods

### Data Source

Discharge rates from 2001–2007 were derived from the Nationwide Inpatient Sample (NIS). The NIS is the largest database of all-payer hospital inpatient stays in the United States (US), containing data from urban, suburban, and rural hospitals across multiple states, approximating a 20-percent stratified sample of US hospitals. [23] It is a joint federal, state, and industry-sponsored project maintained by the Agency for Healthcare Research and Quality. This data is publicly available, and as such was not subject to informed consent for this study nor institutional review board review. Hospitals and states varied over the time period analyzed. Each discharge reported in the NIS is associated with up to 15 International Classification of Diseases, 9^th^ edition (ICD-9) codes, with the first and second recognized as the primary discharge diagnoses and the third through fifteenth as the secondary discharge diagnoses.

To approximate UV light exposure, we used the daily issued UV index forecast from the National Weather Service Climate Prediction Center from 2001–2004, ftp://ftp.cpc.ncep.noaa.gov/long/uv/cities. The issued UV index for each city measures the expected UV radiation when the sun reaches its zenith, and incorporates the elevation of the sun, the amount of ozone, and cloud coverage. This serves as a forecast, ranging from 0 (equivalent to nighttime) to 16 (maximum UV exposure as seen on a clear day in high elevation tropical areas), alerting the public as to the risk of severe burns and long-term skin cancer risk from overexposure. The issued UV index is a forecast and not the actual observed UV index. This forecast is continuously monitored and validated. Issued UV index is reported by city; data were captured for cities within states that were included in the NIS analysis.

### Patient Selection

We identified patients of all ages with IBD-related discharges within the NIS database for each year. This was done by selecting patients with a primary ICD-9 code of CD (555.x) or UC (556.x), or a secondary ICD-9 code of UC or CD and a primary ICD-9 code of an IBD-related complication. [24] IBD related complications included: fistula or intra-abdominal abscess (537.4, 565.1, 567.x, 569.5, 569.81, 569.83, 596.1, 619.1); structuring disease (560.9, 569.2, 537.3); intestinal obstruction (560.x, 568.0); perianal abscess (566.x, 569.4x); gastrointestinal hemorrhage (578.1, 578.9, 569.3); hypovolemia (276.5x); electrolyte imbalance (276.1, 276.8, 275.2, 275.3); anemia (280.x, 285.1, 285.9); or malnutrition (260, 261, 262, 263.x). For every patient identified, the month and state of discharge were recorded. The total number of discharges per state per month was also determined to calculate discharge rates. Additionally, the age, race, payer status, length of stay (LOS), number of procedures (including endoscopy, surgery), and mortality were recorded for each identified patient.

The NIS provides a weighted multiplier for each discharge to facilitate national estimates over multiple years. This number is unique to each hospital, and is based on the American Hospital Association annual survey that incorporates discharge rates. The weight was recorded for each discharge. We analyzed weighted data to control for variation between hospitals and differences in composition of hospitals in the database between years.

### States

States included in analysis were divided into two geographical regions: northern states defined as at or above the 40^th^ parallel and southern states defined as at or below the 35^th^ parallel. We included all states meeting geographical inclusion criteria in the NIS database. Not all eligible states were represented in the NIS database during the years included. Data was abstracted for UV index analysis only from states included in the NIS database and meeting geographical inclusion criteria as above the 40^th^ parallel or below the 35^th^ parallel. Cities/states included for UV analysis were: Little Rock, AR; Phoenix, AZ; Atlanta, GA; Chicago, IL; Boston, MA; Hartford, CT; Des Moines, IA; Detroit, MI; Minneapolis, MN; Raleigh, NC; Concord, NH; Oklahoma City, OK; Portland, OR; Providence, RI; Charleston, SC; Burlington, VT; Seattle, WA; Milwaukee, WI.

### Statistical Analysis

The monthly discharge rates per state were calculated by dividing the IBD-related discharge rate by the total discharge rate for each month. Rates were calculated using weighted discharge data. Within each northern and southern region, the mean discharge rates in the winter (January, February, and March) and summer (July, August, and September) were calculated. These months were chosen as they have been associated with the nadir and apex of vitamin D levels seen in the United States. [25] To assess geographical variation, an odds ratio (OR) was calculated comparing southern states to northern states for each season for weighted rates. To assess seasonal variation, the OR was calculated comparing summer to winter within each region, again using weighted rates. Demographic data and hospital-related factors were analyzed by season and region per year by all IBD, UC only, and CD only with a p ≤ 0.05 considered significant. Chisquare analysis was used to perform the comparisons between groups. Years were analyzed individually and were not combined for analysis due to variability in included states as well as reporting hospitals from year to year.

We performed univariate and multivariate logistic regression models to study the outcome of hospital discharges, length of stay (LOS), procedures, and death in all IBD patients as well as hospital discharges individually in patients with a diagnosis of Crohn’s disease and ulcerative colitis. Length of stay was dichotomized to less than equal to 5 days versus more than 5 days. Five days was chosen based on data showing hospitalized with IBD who are readmitted within 30 days have a mean length of stay of 5.5 days versus 4.3 days for patients not readmitted. [26] Variables which had a p-value <0.1 in the univariate analysis were used in the multivariate regression models. In the multivariate analysis, region (north vs. south) was the primary predictor variable and outcomes were assessed controlling for race, age, payer status, and season for each of the years using weighted data. P-value of ≤ 0.05 was considered statistically significant.

Mean monthly issued UV index for each city was calculated by averaging daily issued UV index forecasts per month. Months where UV index data were unavailable and/or the NIS discharge rates were unavailable. If UV index was available for multiple cities within each state, the state was excluded from this analysis due to our inability to accurately distinguish data from the NIS at the city-level. Each value was plotted and analyzed using the Spearman rank correlation coefficient.

## Results

Utilizing weighted data from the NIS, we identified 76,608 discharges of patients with UC and 143,495 with CD in states either at or above the 40^th^ parallel or below the 35^th^ parallel for years 2001–2007. Demographic data including age, race, and payer status as well as admission-related data including hospital length of stay (five days or less versus more than five days), IBD-related surgical procedures, and mortality were captured (Table 1). Patients in the north were slightly older than patients in the south. The south had a higher percentage of black patients as well as a lower percentage of private insurance compared to north.

**Table 1:** Demographic data for patients included in the study from 2001–2007. Data is for weighted samples.

	Ulcerative Colitis			Crohn’s disease		
	Northern States(weighted n = 48,982)	Southern States(weighted n = 27,626)	P-value	Northern States(weighted n = 90,979)	Southern States(weighted n = 52,516)	P-value
Age at admission (years, range)	47.7 (0–97)	46.3 (0–97)	< 0.001	43.3 (0–100)	42.5 (0–97)	< 0.001
Race: White (%)	83.2	72.5	< 0.001	85.3	77.0	< 0.001
Race: Black (%)	6.9	11.8		8.1	14.9	
Payer: Medicare %)	27.8	27.1	< 0.001	85.3	77.0	< 0.001
Payer: Medicaid (%)	9.7	9.0		12.9	11.7	
Payer: Private insurance (%)	59.2	56.1		61.7	54.6	
Payer: Self pay (%)	3.3	7.8		3.5	9.3	
Length of stay > 5 days (%)	41.6	38.3	< 0.001	32.8	33.0	0.866
Any IBD related surgical procedure (%)	60.2	63.9	< 0.001	42.3	44.2	0.002
Died during hospitalization (%)	1.1	0.9	0.196	0.5	0.5	0.602

### Geographic Discharge Rates

Geographic discharge rate variability was assessed by comparing the mean discharge rates of southern regions to northern regions within summer and winter seasons. In all years analyzed, the mean discharge rates were significantly lower in the southern states compared to the northern states in both summer and winter seasons. This was true for all IBD patients, as well separately for UC or CD only patients (Table 2).

**Table 2:** Discharge OR comparing seasons within each region as well as geography within each season. For each year, the OR were calculated for all IBD, UC only, and CD only.

		Seasonal variations in IBD discharges		Geographical variations in IBD discharges	
		Odds Ratios of summer compared to winter discharges		Odds Ratio of southern compared to northern discharges	
		Northern States	Southern States	Summer	Winter
2001	Total	1.051 (1.022–1.081)	0.963 (0.925–1.003)	0.632 (0.610–0.655)	0.579 (0.559–0.599)
	UC	1.041 (0.993–1.091)	0.858 (0.800–0.921)	0.613 (0.578–0.651)	0.506 (0.476–0.537)
	CD	1.057 (1.021–1.095)	1.020 (0.970–1.071)	0.642 (0.615–0.671)	0.620 (0.594–0.646)
2002	Total	0.945 (0.883–1.010)	0.918 (0.839–1.005)	0.709 (0.656–0.766)	0.689 (0.635–0.747)
	UC	0.966 (0.861–1.083)	0.915 (0.787–1.064)	0.755 (0.662–0.861)	0.715 (0.624–0.820)
	CD	0.934 (0.860–1.014)	0.920 (0.822–1.029)	0.685 (0.623–0.755)	0.675 (0.611–0.746)
2003	Total	0.942 (0.914–0.971)	1.029 (0.990–1.069)	0.670 (0.647–0.693)	0.732 (0.707–0.757)
	UC	0.891 (0.847–0.937)	1.108 (1.038–1.183)	0.605 (0.570–0.641)	0.752 (0.710–0.797)
	CD	0.972 (0.936–1.009)	0.990 (0.945–1.038)	0.708 (0.678–0.738)	0.721 (0.691–0.752)
2004	Total	0.980 (0.924–1.040)	0.919 (0.847–0.998)	0.633 (0.590–0.679)	0.593 (0.552–0.638)
	UC	1.026 (0.928–1.134)	0.890 (0.773–1.024)	0.641 (.568–0.723)	0.556 (0.491–0.629)
	CD	0.957 (0.890–1.030)	0.935 (0.845–1.033)	0.629 (0.577–0.686)	0.614 (0.562–0.671)
2005	Total	0.968 (0.942–0.996)	0.940 (0.909–0.972)	0.754 (0.732–0.778)	0.733 (0.710–0.756)
	UC	0.942 (0.898–0.988)	0.980 (0.925–1.039)	0.708 (0.671–0.746)	0.736 (0.698–0.777)
	CD	0.982 (0.949–1.016)	0.921 (0.884–0.960)	0.779 (0.751–0.809)	0.731 (0.704–0.759)
2006	Total	0.968 (0.942–0.996)	0.940 (0.909–0.972)	0.754 (0.732–0.778)	0.733 (0.710–0.756)
	UC	0.942 (0.898–0.988)	0.980 (0.925–1.039)	0.708 (0.671–0.746)	0.736 (0.698–0.777)
	CD	0.982 (0.949–1.016)	0.921 (0.884–0.960)	0.779 (0.751–0.809)	0.731 (0.704–0.759)
2007	Total	0.906 (0.882–0.930)	0.899 (0.869–0.929)	0.757 (0.735–0.780)	0.751 (0.729–0.775)
	UC	0.901 (0.862–0.942)	0.862 (0.815–0.912)	0.781 (0.743–0.820)	0.747 (0.709–0.786)
	CD	0.908 0.879–0.939)	0.920 (0.882–0.959)	0.744 (0.717–0.772)	0.754 (0.726–0.783)

### Seasonal Discharge Rates

Seasonal discharge rate variability was assessed by comparing the mean discharge rates of summer months compared to winter months within each region. With a few exceptions as noted in the table, rates were either similar or lower in the summer in both the southern and northern states when examining all IBD patients as well as CD or UC groups individually (Table 2).

### Multivariate Analysis

In the multivariate model, there was a protective effect for the southern states compared to northern states for all IBD discharges as well as either UC or CD alone. Additionally, there was a protective effect in the southern states with respect to LOS. Effects of latitude on inpatient procedures and deaths during hospitalization were not consistent across the years studied (Table 3).

**Table 3:** Multivariate analysis comparing south versus north. Variables included in the multivariate analysis included race, age, and payer status.

	OR	95% CI
All IBD		
2001	0.616	0.597–0.634
2002	0.812	0.787–0.838
2003	0.653	0.634–0.672
2004	0.609	0.593–0.626
2005	0.717	0.699–0.736
2006	0.699	0.682–0.718
2007	0.714	0.696–0.733
UC		
2001	0.571	0.542–0.602
2002	0.866	0.821–0.913
2003	0.613	0.583–0.644
2004	0.627	0.599–0.656
2005	0.697	0.667–0.730
2006	0.752	0.721–0.785
2007	0.739	0.708–0.772
CD		
2001	0.639	0.616–0.663
2002	0.784	0.753–0.815
2003	0.676	0.652–0.701
2004	0.600	0.580–0.620
2005	0.727	0.704–0.751
2006	0.670	0.649–0.693
2007	0.700	0.677–0.723
LOS		
2001	0.865	0.862–0.869
2002	0.989	0.985–0.993
2003	0.977	0.973–0.981
2004	0.898	0.895–0.902
2005	0.893	0.890–0.896
2006	0.911	0.908–0.915
2007	0.949	0.946–0.953
Procedure		
2001	1.044	1.038–1.050
2002	1.211	1.204–1.219
2003	1.058	1.053–1.064
2004	1.033	1.027–1.038
2005	0.926	0.922–0.931
2006	0.900	0.895–0.904
2007	0.937	0.933–0.942
Death		
2001	0.985	0.975–0.996
2002	1.112	1.099–1.125
2003	0.957	0.947–0.968
2004	0.924	0.915–0.934
2005	0.924	0.933–0.952
2006	1.058	1.047–1.070
2007	1.046	1.034–1.057

### Ultraviolet Index

**Fig 1:**
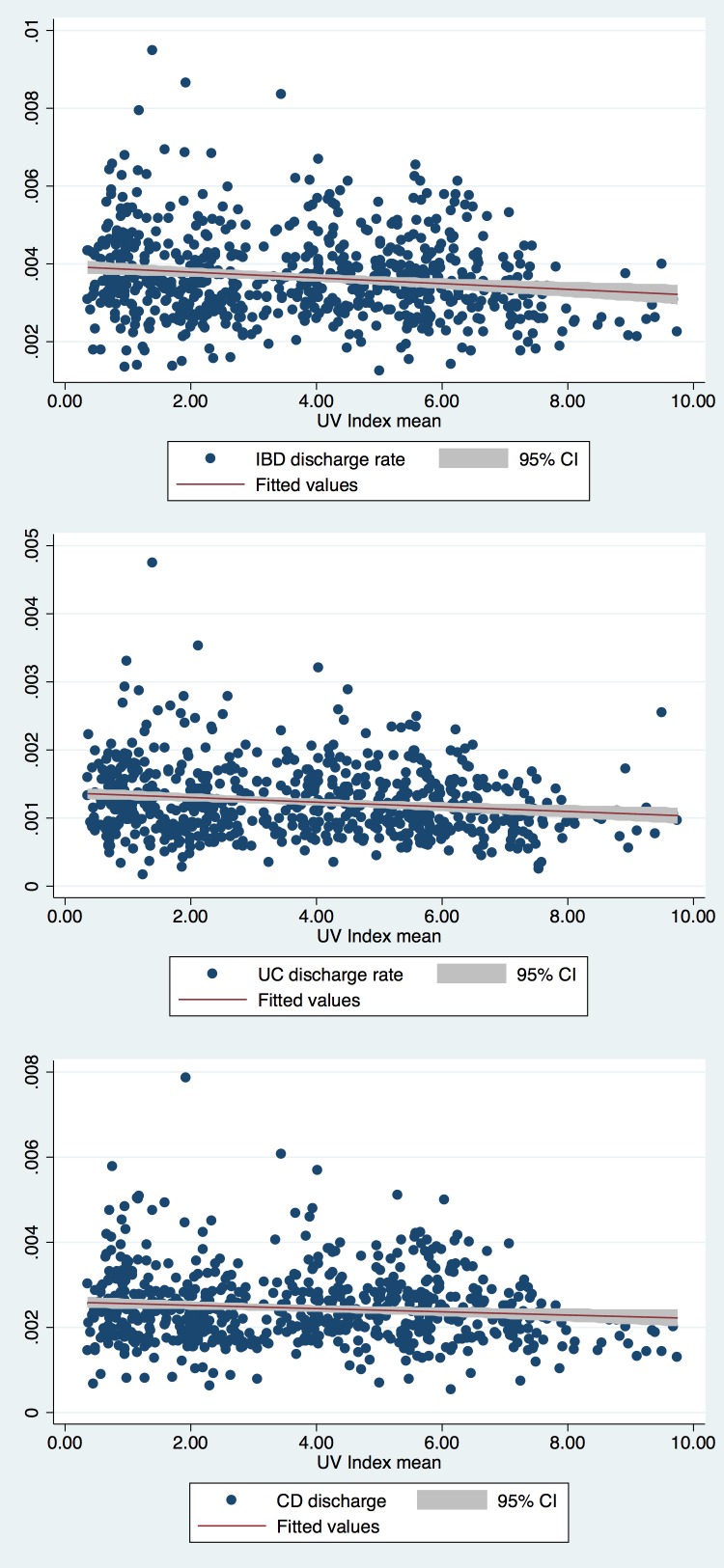
Monthly discharge rates with associated mean UV index values for 2001–2004 for included northern and southern states. (a) Discharge rates for all IBD (p<0.001), (b) discharge rates for UC only (p<0.001), (c) discharge rates for CD only (p = 0.018).

The UV index by month from 2001–2004 for cities both in the southern and northern regions were compared. Missing data included Charleston, SC (all of 2001). We found a small but statistically significant inverse correlation between lower discharge rates and higher UV index when examining all IBD patients (p<0.001) as well as UC (p<0.001) and CD patients (p = 0.018) individually (Fig 1).

## Discussion

Using the NIS database for years 2001–2007, we demonstrated a consistent pattern of increased IBD-related hospitalization rates in northern states as compared to states in the southern US. This finding was observed for both UC and CD. There was not a consistently observed difference in hospitalization rates comparing summer to winter months within either region, other than in 2007 where both regions showed summer having a lower hospitalization rate compared to winter as well as for UC and CD. This was not otherwise consistently observed.

Although there are several works examining the impact of geography on IBD incidence, to our knowledge, this is only the second study evaluating latitudinal gradients of disease activity in IBD. The first study, published in 1991, measured hospital discharges of all U.S. Medicare patients during 1986–1987 with an IBD-related code (either primary or secondary), and found that there were increased numbers of hospitalizations carrying an IBD diagnosis in northern states as compared to southern states. [4] The results of this study are limited as they assessed only patient’s over 65, and were not able to differentiate between primary admissions related to IBD vs. secondary causes not related to disease activity. We have extended this analysis by showing that patients, regardless of age, are hospitalized in northern latitudes at higher rates for indications primarily related to their IBD.

There is a growing body of evidence suggesting a relationship between IBD and the north-south gradient. Seasonal variation is less established. Previous analyses have identified seasonal variation in other autoimmune diseases, including Behcet’s and lupus, which is consistent with the finding that the immune system has a proinflammatory transcriptomic profile in the winter. In contrast to these studies as well as those that have investigated seasonal variation in infection such as influenza, we did not identify a consistent association between season and IBD admissions. [27–30] This is in agreement with other prior studies [17,31] but not all [19,21,22,32]. Some other studies have shown a relationship between latitude and incidence for IBD [1–3,5–7]; our study is the first to confirm a prior study showing there is a hospitalization risk as well. [4]

The geographical skew observed in IBD discharge rates is in-line with the increased incidence of IBD observed in northern compared to southern latitudes. [3–8] As such, the increase in hospitalizations observed in northern states may reflect a higher disease prevalence, thus leading to an expected greater rate of hospitalizations compared to southern regions. Alternatively, the geographical variation in IBD-related admissions may suggest significant differences in factors associated with disease activity in northern latitudes. Although the reason for this variation is not known, it may be related to several environmental factors such as sunlight exposure, infectious diseases, sanitation, environmental toxins, economics, or genetic clustering. Unfortunately, a major limitation in our study is the inability to accurately measure potential causal or co-morbid factors at the individual level including presenting symptoms, laboratory results, prior medical problems, or family history that are not available in the NIS database.

Sunlight exposure and its relation to vitamin D, one of the leading hypotheses of an environmental exposure related to disease activity, is not fully supported by the results of this study. Although IBD admissions were higher in northern latitudes and an inverse association between UV index and discharge rates was observed, we failed to find consistent differences in hospitalization rates by season when controlling for latitude. There was one exception, with 2007 showing a protective effect during summer for both regions as well as both UC and CD. The reasoning for this protective effect in 2007 remains unclear. It is possible that this effect is seen as the result of more robust data, as 2007 had the most states included for analysis. However, we are uncertain of this significance, as states were slowly added each year to the NIS dataset, and we did not see any significant change in data in other years, including 2005 and 2006 which only differed by one state (Maine) from 2007. It would be of interest in future studies to investigate more recent years, assessing potential trends in seasonal variation.

Furthermore, residents of southern states were more likely to have longer hospital stays and more likely to undergo surgery than those in northern states. While these findings may reflect other factors, such as demographic differences, regional practice patterns, as well as additional comorbidities in the patient population, they also suggest more severe disease activity in the southern states. We speculate that if sunlight were linked to disease activity, the data would not only show a geographic latitudinal shift, but also a more substantial and consistent seasonal change, with a more pronounced decrease in hospitalization rates in the summer compared to winter. It is possible that our choice of months lead to a beta-error, and that there is a delay in sunlight exposure on disease activity. As such, looking at spring or fall months might show greater differences. However, the lack of seasonal variation observed in the current study is in line with published data examining the incidence of UC or CD. [18] Other potential confounders for the lack of seasonal rate change include travel and seasonal changes in residence (such as snowbirds moving from northern regions to southern regions for a period of time during the winter months).

There are several limitations with our study. While we utilized the NIS, which is the largest all-payer database representing a 20-percent stratified sample of US hospitals, we are unable to fully account for hospital-level data in our analysis. As such, reporting from academic hospitals and IBD-referral centers that tend to draw both primary admissions as well as transfers of patients with IBD-related illness might be unpredictably over- or under-reported. This may contribute to the northern predominance, including density of IBD-referral centers as well as patient migration. The NIS normalizes its raw data to account for under or over collection of data across regions to mirror the true population. [23] While this normalization helps to prevent reporting (or non-reporting) skewed data from regional IBD-centers, both of these represent limitations to the dataset and could potentially confound our findings. A second limitation is that regional practice patterns and demographics may influence the admission rates. It is interesting that patients in the southern region, while having lower rates of hospitalizations, also have longer stays and potentially more IBD-related surgical procedures. Demographical differences may account for this as there is a larger proportion of African American patients, as well as government payer patients in the southern states. Additionally, we were unable to capture disease activity, and as such were unable to determine the severity of patients hospitalized to compare practice patterns. Nonetheless, we believe the need for hospitalization is an objective marker for more severe disease activity, but concede that regional variation in demographics and practice patterns could also contribute to the latitude dependent differences as well. We also did not capture vitamin D levels in the NIS database. There has not been an analysis that we are aware of looking at the accuracy of vitamin d deficiency reporting within the NIS. We question the accuracy of this data due to reporting bias, and as such were not able to relate vitamin D levels to UV exposure. The relationship between UV exposure and discharge rates is also limited by inability to control for altitude, both within state as well as accounting for regional variation in patients seen at tertiary-care and IBD referral centers.

In conclusion, using the NIS database, we identified a significant increased rate of IBD-related hospitalizations in northern compared to southern states in the US. These observed latitudinal differences in IBD-related discharges are unlikely to be fully explained by differences in sunlight and UV exposure. We were unable to analyze individual patient data, however, limiting our ability to imply causation. In this regard, future studies focusing on other environmental factors that could contribute to these geographic differences might illuminate other key disease aggravating factors in patients with IBD.

## Supporting Information

S1 Data Data for Tables 1–3. (PDF)

S2 Data UV index data. (XLSX)
