# Supplementary material for: Northern Latitude but Not Season Is Associated with Increased Rates of Hospitalizations Related to Inflammatory Bowel Disease: Results of a Multi-Year Analysis of a National Cohort
Source: PLoS One. 2016 Aug 31;11(8):e0161523. doi: 10.1371/journal.pone.0161523 (PMC5007007; doi:10.1371/journal.pone.0161523)
Supplement: S1 Data — (PDF) [file pone.0161523.s001.pdf]

```
      name:  <unnamed>
       log:  U:\RKunnavakkam\Adam_Stein\output.smcl
  log type:  smcl
 opened on:  19 Feb 2013, 16:06:26

 1 . // YEAR 2001
   unrecognized command:  / invalid command name
   r(199);

 2 . //**********************************************************************
   unrecognized command:  / invalid command name
   r(199);

 3 .
 4 . // PART1. UC or CD cases
   unrecognized command:  / invalid command name
   r(199);

 5 .
 6 . //**********************************************************************
   unrecognized command:  / invalid command name
   r(199);

 7 .
 8 .
 9 .
10 . svyset hospid [pweight=discwt], strata (nis_stratum) singleunit(centered)

      pweight: discwt
          VCE: linearized
  Single unit: centered
     Strata 1: nis_stratum
         SU 1: hospid
        FPC 1: <zero>

11 .
12 . svy: logistic uc_cd_new region season_new
   (running logistic on estimation sample)

   Survey: Logistic regression

   Number of strata   =        58                  Number of obs      =    1809449
   Number of PSUs     =       500                  Population size    =  9049484.6
                                                   Design df          =        442
                                                   F(   2,    441)    =      10.90
                                                   Prob > F           =     0.0000
```

uc_cd_new	Odds Ratio	Linearized Std. Err.	t	P>|t|	[95% Conf.	Interval]
region	1.713094	.1973467	4.67	0.000	1.366012	2.148365
season_new	.9997766	.0458608	-0.00	0.996	.9135877	1.094097

_cons	**388.6565**	**86.35966**	**26.83**	**0.000**	**251.1361**	**601.482**

Note: strata with single sampling unit centered at overall mean.

```
13 .
14 . svy: logistic uc_cd_new i.region
(running logistic on estimation sample)
```

Survey: Logistic regression

Number of strata =	**58**		Number of obs =	**4265087**
Number of PSUs =	**557**		Population size =	**21198251**
			Design df =	**499**
			F(**1**, **499**) =	**17.38**
			Prob > F =	**0.0000**

uc_cd_new	Odds Ratio	Linearized Std. Err.	t	P>|t|	[95% Conf.	Interval]
2.region	**1.629813**	**.1909762**	**4.17**	**0.000**	**1.294656**	**2.051735**
_cons	**651.2202**	**70.31693**	**60.00**	**0.000**	**526.7372**	**805.122**

Note: strata with single sampling unit centered at overall mean.

```
15 .
16 . margins region
```

Adjusted predictions Number of obs = **4265087**
Model VCE : **Linearized**

Expression : **Pr(uc_cd_new), predict()**

	Margin	Delta-method Std. Err.	z	P>|z|	[95% Conf.	Interval]
region						
1	**.9984668**	**.0001653**	**6040.35**	**0.000**	**.9981428**	**.9987908**
2	**.9990587**	**.0000427**	**2.3e+04**	**0.000**	**.9989749**	**.9991425**

```
17 .
18 . margins region, post
```

Adjusted predictions Number of obs = **4265087**
Model VCE : **Linearized**

Expression : **Pr(uc_cd_new), predict()**

	Margin	Delta-method Std. Err.	z	P>|z|	[95% Conf.	Interval]

region						
1	**.9984668**	**.0001653**	**6040.35**	**0.000**	**.9981428**	**.9987908**
2	**.9990587**	**.0000427**	**2.3e+04**	**0.000**	**.9989749**	**.9991425**

```
19 .
20 . // OVERALL INCIDENCE RATE UC or CD cases : North REGION
   unrecognized command:  / invalid command name
   r(199);

21 .
22 . lincom 1-1.region

    ( 1)  - 1bn.region = -1
```

	Coef.	Std. Err.	z	P>|z|	[95% Conf.	Interval]
(1)	**.0015332**	**.0001653**	**9.28**	**0.000**	**.0012092**	**.0018572**

```
23 .
24 . // OVERALL INCIDENCE RATE UC or CD cases : South REGION
   unrecognized command:  / invalid command name
   r(199);

25 .
26 . lincom 1-2.region

    ( 1)  - 2.region = -1
```

	Coef.	Std. Err.	z	P>|z|	[95% Conf.	Interval]
(1)	**.0009413**	**.0000427**	**22.02**	**0.000**	**.0008575**	**.0010251**

```
27 .
28 . // This gives results for within region comparing season1 and season 2
   unrecognized command:  / invalid command name
   r(199);

29 .
30 . //Comparison within North Region between Winter and Summer Season (Reference: WInter month)
   unrecognized command:  / invalid command name
   r(199);

31 .
32 . svy: logistic uc_cd_new  r1s
   (running logistic on estimation sample)

   Survey: Logistic regression

   Number of strata   =        36                  Number of obs      =    895633
```

```
Number of PSUs     =       247                 Population size    = 4645214.8
                                               Design df          =       211
                                               F(   1,    211)    =      0.02
                                               Prob > F           =    0.8954
```

uc_cd_new	Odds Ratio	Linearized Std. Err.	t	P>|t|	[95% Conf.	Interval]
r1s	1.007341	.0559517	0.13	0.895	.9028686	1.123901
_cons	658.2925	88.65748	48.19	0.000	504.8002	858.4565

Note: strata with single sampling unit centered at overall mean.

```
33 .
34 . //Comparison within South Region between Winter and Summer Season (Reference: WInter month
   unrecognized command:  / invalid command name
   r(199);

35 .
36 . svy: logistic uc_cd_new   r2s
   (running logistic on estimation sample)

   Survey: Logistic regression

   Number of strata   =        31                 Number of obs      =    913816
   Number of PSUs     =       253                 Population size    = 4404269.8
                                                  Design df          =       222
                                                  F(   1,    222)    =      0.03
                                                  Prob > F           =    0.8624
```

uc_cd_new	Odds Ratio	Linearized Std. Err.	t	P>|t|	[95% Conf.	Interval]
r2s	.9862716	.0785847	-0.17	0.862	.8429509	1.15396
_cons	1164.065	158.1731	51.96	0.000	890.5983	1521.502

Note: strata with single sampling unit centered at overall mean.

```
37 .
38 .
39 .
40 . //*******************************************************************************************
   unrecognized command:  / invalid command name
   r(199);

41 .
42 . // PART2. UC cases only
   unrecognized command:  / invalid command name
   r(199);

43 .
44 . //******************************************************************************************
```

```
unrecognized command:  / invalid command name
r(199);

45 .
46 . svy: logistic uc_cases_new i.region season_new
(running logistic on estimation sample)

Survey: Logistic regression

Number of strata   =        58                  Number of obs      =    1809449
Number of PSUs     =       500                  Population size    = 9049484.6
                                                Design df          =        442
                                                F(   2,    441)    =      10.92
                                                Prob > F           =     0.0000
```

uc_cases_new	Odds Ratio	Linearized Std. Err.	t	P>|t|	[95% Conf.	Interval]
2.region	2.038883	.3198578	4.54	0.000	1.497925	2.775202
season_new	1.010277	.0836666	0.12	0.902	.8585275	1.188849
_cons	1777.614	373.4634	35.62	0.000	1176.292	2686.331

```
Note: strata with single sampling unit centered at overall mean.

47 .
48 . margins region

Predictive margins                              Number of obs   =    1809449
Model VCE    : Linearized

Expression   : Pr(uc_cases_new), predict()
```

	Margin	Delta-method Std. Err.	z	P>|z|	[95% Conf.	Interval]
region						
1	.9994463	.000077	1.3e+04	0.000	.9992954	.9995973
2	.9997284	.0000192	5.2e+04	0.000	.9996908	.9997659

```
49 .
50 . margins region, post

Predictive margins                              Number of obs   =    1809449
Model VCE    : Linearized

Expression   : Pr(uc_cases_new), predict()
```

	Margin	Delta-method Std. Err.	z	P>|z|	[95% Conf.	Interval]

```
      region |
          1  |   .9994463    .000077  1.3e+04   0.000     .9992954    .9995973
          2  |   .9997284   .0000192  5.2e+04   0.000     .9996908    .9997659
------------------------------------------------------------------------------

51 .
52 . // OVERALL INCIDENCE RATE UC only: North REGION
   unrecognized command:  / invalid command name
   r(199);

53 .
54 . lincom 1-1.region

 ( 1)  - 1bn.region = -1

------------------------------------------------------------------------------
             |      Coef.   Std. Err.      z    P>|z|     [95% Conf. Interval]
-------------+----------------------------------------------------------------
         (1) |   .0005537    .000077     7.19   0.000     .0004027    .0007046
------------------------------------------------------------------------------

55 .
56 . // OVERALL INCIDENCE RATE UC only : South REGION
   unrecognized command:  / invalid command name
   r(199);

57 .
58 . lincom 1-2.region

 ( 1)  - 2.region = -1

------------------------------------------------------------------------------
             |      Coef.   Std. Err.      z    P>|z|     [95% Conf. Interval]
-------------+----------------------------------------------------------------
         (1) |   .0002716   .0000192    14.17   0.000     .0002341    .0003092
------------------------------------------------------------------------------

59 .
60 .
61 .
62 . //Comparison within North Region between Winter and Summer Season (Reference: WInter month)
   unrecognized command:  / invalid command name
   r(199);

63 .
64 . svy: logistic uc_cases_new  r1s
   (running logistic on estimation sample)

   Survey: Logistic regression

   Number of strata   =        36                 Number of obs      =    895633
   Number of PSUs     =       247                 Population size    = 4645214.8
                                                  Design df          =       211
                                                  F(   1,    211)    =      0.67
```

```
                                                Prob > F          =    0.4133

------------------------------------------------------------------------------
             |             Linearized
 uc_cases_new | Odds Ratio   Std. Err.      t    P>|t|     [95% Conf. Interval]
-------------+----------------------------------------------------------------
          r1s |   1.082486   .1046768     0.82   0.413     .8946141    1.309811
        _cons |   1603.258   357.6727    33.08   0.000     1032.791    2488.826
------------------------------------------------------------------------------
Note: strata with single sampling unit centered at overall mean.

65 .
66 . //Comparison within South Region between Winter and Summer Season (Reference: WInter month)
unrecognized command:  / invalid command name
r(199);

67 .
68 . svy: logistic uc_cases_new  r2s
(running logistic on estimation sample)

Survey: Logistic regression

Number of strata   =        31                  Number of obs      =    913816
Number of PSUs     =       253                  Population size    = 4404269.8
                                                Design df          =       222
                                                F(   1,    222)    =      0.95
                                                Prob > F           =    0.3310

------------------------------------------------------------------------------
             |             Linearized
 uc_cases_new | Odds Ratio   Std. Err.      t    P>|t|     [95% Conf. Interval]
-------------+----------------------------------------------------------------
          r2s |   .8708695   .1235973    -0.97   0.331     .6583926    1.151917
        _cons |    4536.89   1018.426    37.51   0.000     2914.983    7061.233
------------------------------------------------------------------------------
Note: strata with single sampling unit centered at overall mean.

69 .
70 .
71 .
72 . //*****************************************************************************
unrecognized command:  / invalid command name
r(199);

73 .
74 . // PART3. CD Cases only
unrecognized command:  / invalid command name
r(199);

75 .
76 . ********************************************************************************

77 .
78 . svy: logistic cd_cases_new i.region season_new
```

```
(running logistic on estimation sample)

Survey: Logistic regression

Number of strata   =        58                  Number of obs      =   1809449
Number of PSUs     =       500                  Population size    = 9049484.6
                                                Design df          =       442
                                                F(   2,    441)    =      8.86
                                                Prob > F           =    0.0002
```

cd_cases_new	Odds Ratio	Linearized Std. Err.	t	P>|t|	[95% Conf.	Interval]
2.region	1.550355	.1640464	4.14	0.000	1.259263	1.908736
season_new	1.010421	.0531303	0.20	0.844	.9112161	1.120427
_cons	975.6526	107.852	62.27	0.000	785.1309	1212.407

Note: strata with single sampling unit centered at overall mean.

```
79 .
80 . svy: logistic cd_cases_new i.region
   (running logistic on estimation sample)

   Survey: Logistic regression

   Number of strata   =        58                  Number of obs      =   4265087
   Number of PSUs     =       557                  Population size    =  21198251
                                                   Design df          =       499
                                                   F(   1,    499)    =     13.46
                                                   Prob > F           =    0.0003
```

cd_cases_new	Odds Ratio	Linearized Std. Err.	t	P>|t|	[95% Conf.	Interval]
2.region	1.501617	.1663744	3.67	0.000	1.207868	1.866805
_cons	979.1612	97.53338	69.14	0.000	805.1201	1190.825

Note: strata with single sampling unit centered at overall mean.

```
81 .
82 . margins region

   Adjusted predictions                              Number of obs    =    4265087
   Model VCE    : Linearized

   Expression   : Pr(cd_cases_new), predict()
```

	Margin	Delta-method Std. Err.	z	P>|z|	[95% Conf.	Interval]
region						

1	**.9989798**	**.0001015**	**9840.08**	**0.000**	**.9987808**	**.9991787**
2	**.9993203**	**.000033**	**3.0e+04**	**0.000**	**.9992557**	**.999385**

```
83 .
84 . margins region, post

   Adjusted predictions                              Number of obs   =    4265087
   Model VCE    : Linearized

   Expression   : Pr(cd_cases_new), predict()
```

	Margin	Delta-method Std. Err.	z	P>|z|	[95% Conf.	Interval]
region						
1	**.9989798**	**.0001015**	**9840.08**	**0.000**	**.9987808**	**.9991787**
2	**.9993203**	**.000033**	**3.0e+04**	**0.000**	**.9992557**	**.999385**

```
85 .
86 . // OVERALL INCIDENCE RATE CD only: North REGION
   unrecognized command:  / invalid command name
   r(199);

87 .
88 . lincom 1-1.region

    ( 1)  - 1bn.region = -1
```

	Coef.	Std. Err.	z	P>|z|	[95% Conf.	Interval]
(1)	**.0010202**	**.0001015**	**10.05**	**0.000**	**.0008213**	**.0012192**

```
89 .
90 . // OVERALL INCIDENCE RATE CD only: South REGION
   unrecognized command:  / invalid command name
   r(199);

91 .
92 . lincom 1-2.region

    ( 1)  - 2.region = -1
```

	Coef.	Std. Err.	z	P>|z|	[95% Conf.	Interval]
(1)	**.0006797**	**.000033**	**20.61**	**0.000**	**.000615**	**.0007443**

```
93 .
```

```
 94 .
 95 .
 96 . //Comparison within North Region between Winter and Summer Season (Reference: WInter month)
    unrecognized command:  / invalid command name
    r(199);

 97 .
 98 . svy: logistic cd_cases_new  r1s
    (running logistic on estimation sample)

    Survey: Logistic regression

    Number of strata   =        36                Number of obs      =    895633
    Number of PSUs     =       247                Population size    = 4645214.8
                                                  Design df          =       211
                                                  F(   1,    211)    =      0.01
                                                  Prob > F           =    0.9091
```

cd_cases_new	Odds Ratio	Linearized Std. Err.	t	P>|t|	[95% Conf.	Interval]
r1s	.9922869	.0672432	-0.11	0.909	.8682047	1.134103
_cons	1002.633	126.3769	54.82	0.000	782.0477	1285.436

```
    Note: strata with single sampling unit centered at overall mean.

 99 .
100 . //Comparison within South Region between Winter and Summer Season (Reference: WInter month)
    unrecognized command:  / invalid command name
    r(199);

101 .
102 . svy: logistic cd_cases_new  r2s
    (running logistic on estimation sample)

    Survey: Logistic regression

    Number of strata   =        31                Number of obs      =    913816
    Number of PSUs     =       253                Population size    = 4404269.8
                                                  Design df          =       222
                                                  F(   1,    222)    =      0.24
                                                  Prob > F           =    0.6281
```

cd_cases_new	Odds Ratio	Linearized Std. Err.	t	P>|t|	[95% Conf.	Interval]
r2s	1.040773	.0857514	0.49	0.628	.8847883	1.224257
_cons	1447.35	208.6251	50.49	0.000	1089.447	1922.829

```
    Note: strata with single sampling unit centered at overall mean.

103 . clear
```

```
104 . // YEAR 2002
    unrecognized command:  / invalid command name
    r(199);

105 . use "U:\RKunnavakkam\Adam_Stein\2002\UC_CD_2002_req.dta", clear

106 . //*******************************************************************
    unrecognized command:  / invalid command name
    r(199);

107 .
108 . // PART1. UC or CD cases
    unrecognized command:  / invalid command name
    r(199);

109 .
110 . //*******************************************************************
    unrecognized command:  / invalid command name
    r(199);

111 .
112 .
113 .
114 . svyset hospid [pweight=discwt], strata (nis_stratum) singleunit(centered)

          pweight: discwt
              VCE: linearized
      Single unit: centered
         Strata 1: nis_stratum
             SU 1: hospid
            FPC 1: <zero>

115 .
116 . svy: logistic uc_cd_new region season_new
    (running logistic on estimation sample)

    Survey: Logistic regression

    Number of strata   =        58                Number of obs      =   1718528
    Number of PSUs     =       490                Population size    = 8357508.6
                                                  Design df          =       432
                                                  F(   2,    431)     =      7.79
                                                  Prob > F           =    0.0005
```

uc_cd_new	Odds Ratio	Linearized Std. Err.	t	P>|t|	[95% Conf.	Interval]
region	1.541895	.1696237	3.94	0.000	1.242084	1.914073
season_new	1.005729	.044957	0.13	0.898	.9211382	1.098089
_cons	429.2868	82.62343	31.50	0.000	294.0758	626.6655

Note: strata with single sampling unit centered at overall mean.

```
117 .
118 . svy: logistic uc_cd_new i.region
    (running logistic on estimation sample)

    Survey: Logistic regression

    Number of strata   =        58                  Number of obs      =   4180307
    Number of PSUs     =       543                  Population size    =  20203730
                                                    Design df          =       485
                                                    F(   1,    485)    =     26.52
                                                    Prob > F           =    0.0000
```

uc_cd_new	Odds Ratio	Linearized Std. Err.	t	P>|t|	[95% Conf.	Interval]
2.region	**1.603857**	**.1471172**	**5.15**	**0.000**	**1.339344**	**1.92061**
_cons	**661.4406**	**46.97947**	**91.44**	**0.000**	**575.2838**	**760.5005**

Note: strata with single sampling unit centered at overall mean.

```
119 .
120 . margins region

    Adjusted predictions                              Number of obs    =    4180307
    Model VCE    : Linearized

    Expression   : Pr(uc_cd_new), predict()
```

	Margin	Delta-method Std. Err.	z	P>|z|	[95% Conf.	Interval]
region						
1	**.9984904**	**.0001071**	**9326.74**	**0.000**	**.9982806**	**.9987003**
2	**.9990583**	**.0000553**	**1.8e+04**	**0.000**	**.9989499**	**.9991666**

```
121 .
122 . margins region, post

    Adjusted predictions                              Number of obs    =    4180307
    Model VCE    : Linearized

    Expression   : Pr(uc_cd_new), predict()
```

	Margin	Delta-method Std. Err.	z	P>|z|	[95% Conf.	Interval]
region						
1	**.9984904**	**.0001071**	**9326.74**	**0.000**	**.9982806**	**.9987003**
2	**.9990583**	**.0000553**	**1.8e+04**	**0.000**	**.9989499**	**.9991666**

```
123 .
124 . // OVERALL INCIDENCE RATE UC or CD cases : North REGION
    unrecognized command:  / invalid command name
    r(199);

125 .
126 . lincom 1-1.region

     ( 1)  - 1bn.region = -1
```

	Coef.	Std. Err.	z	P>|z|	[95% Conf.	Interval]
(1)	.0015096	.0001071	14.10	0.000	.0012997	.0017194

```
127 .
128 . // OVERALL INCIDENCE RATE UC or CD cases : South REGION
    unrecognized command:  / invalid command name
    r(199);

129 .
130 . lincom 1-2.region

     ( 1)  - 2.region = -1
```

	Coef.	Std. Err.	z	P>|z|	[95% Conf.	Interval]
(1)	.0009417	.0000553	17.04	0.000	.0008334	.0010501

```
131 .
132 . // This gives results for within region comparing season1 and season 2
    unrecognized command:  / invalid command name
    r(199);

133 .
134 . //Comparison within North Region between Winter and Summer Season (Reference: WInter month)
    unrecognized command:  / invalid command name
    r(199);

135 .
136 . svy: logistic uc_cd_new  r1s
    (running logistic on estimation sample)

    Survey: Logistic regression

    Number of strata   =        36                Number of obs      =    861149
    Number of PSUs     =       244                Population size    = 4345308.2
                                                  Design df          =       208
                                                  F(   1,    208)    =      0.95
```

```
                                                Prob > F          =    0.3312

------------------------------------------------------------------------------
             |             Linearized
   uc_cd_new | Odds Ratio   Std. Err.      t    P>|t|     [95% Conf. Interval]
-------------+----------------------------------------------------------------
         r1s |     1.0588   .0621107     0.97   0.331     .9431678    1.188608
       _cons |   612.9152   71.85878    54.74   0.000     486.4303    772.2895
------------------------------------------------------------------------------
Note: strata with single sampling unit centered at overall mean.

137 .
138 . //Comparison within South Region between Winter and Summer Season (Reference: WInter month
unrecognized command:  / invalid command name
r(199);

139 .
140 . svy: logistic uc_cd_new   r2s
(running logistic on estimation sample)

Survey: Logistic regression

Number of strata   =        26                  Number of obs      =    857379
Number of PSUs     =       246                  Population size    = 4012200.4
                                                Design df          =       220
                                                F(   1,    220)    =      1.38
                                                Prob > F           =    0.2422

------------------------------------------------------------------------------
             |             Linearized
   uc_cd_new | Odds Ratio   Std. Err.      t    P>|t|     [95% Conf. Interval]
-------------+----------------------------------------------------------------
         r2s |   .9230333   .0630397    -1.17   0.242     .8067926    1.056022
       _cons |   1161.585   145.0245    56.53   0.000     908.2185    1485.634
------------------------------------------------------------------------------
Note: strata with single sampling unit centered at overall mean.

141 .
142 .
143 .
144 . //***************************************************************************************
unrecognized command:  / invalid command name
r(199);

145 .
146 . // PART2. UC cases only
unrecognized command:  / invalid command name
r(199);

147 .
148 . //**************************************************************************************
unrecognized command:  / invalid command name
r(199);
```

```
149 .
150 . svy: logistic uc_cases_new i.region season_new
    (running logistic on estimation sample)

    Survey: Logistic regression

    Number of strata   =        58                  Number of obs      =   1718528
    Number of PSUs     =       490                  Population size    = 8357508.6
                                                    Design df          =       432
                                                    F(   2,    431)    =      8.84
                                                    Prob > F           =    0.0002

    ─────────────┬────────────────────────────────────────────────────────────────
                 │             Linearized
    uc_cases_new │ Odds Ratio   Std. Err.      t    P>|t|     [95% Conf. Interval]
    ─────────────┼────────────────────────────────────────────────────────────────
        2.region │    1.79584   .2497609     4.21   0.000     1.366318    2.360387
      season_new │   1.040338     .07194     0.57   0.568     .9081295    1.191793
           _cons │   1733.694   265.3395    48.73   0.000     1283.309    2342.144
    ─────────────┴────────────────────────────────────────────────────────────────
    Note: strata with single sampling unit centered at overall mean.

151 .
152 . margins region

    Predictive margins                                Number of obs    =    1718528
    Model VCE    : Linearized

    Expression   : Pr(uc_cases_new), predict()

    ─────────────┬────────────────────────────────────────────────────────────────
                 │            Delta-method
                 │     Margin   Std. Err.      z    P>|z|     [95% Conf. Interval]
    ─────────────┼────────────────────────────────────────────────────────────────
          region │
               1 │   .9994566   .0000525  1.9e+04   0.000     .9993537    .9995595
               2 │   .9996974   .0000304  3.3e+04   0.000     .9996377     .999757
    ─────────────┴────────────────────────────────────────────────────────────────

153 .
154 . margins region, post

    Predictive margins                                Number of obs    =    1718528
    Model VCE    : Linearized

    Expression   : Pr(uc_cases_new), predict()

    ─────────────┬────────────────────────────────────────────────────────────────
                 │            Delta-method
                 │     Margin   Std. Err.      z    P>|z|     [95% Conf. Interval]
    ─────────────┼────────────────────────────────────────────────────────────────
          region │
               1 │   .9994566   .0000525  1.9e+04   0.000     .9993537    .9995595
               2 │   .9996974   .0000304  3.3e+04   0.000     .9996377     .999757
```

```
155 .
156 . // OVERALL INCIDENCE RATE UC only: North REGION
    unrecognized command:  / invalid command name
    r(199);

157 .
158 . lincom 1-1.region

     ( 1)  - 1bn.region = -1
```

	Coef.	Std. Err.	z	P>|z|	[95% Conf.	Interval]
(1)	.0005434	.0000525	10.35	0.000	.0004405	.0006463

```
159 .
160 . // OVERALL INCIDENCE RATE UC only : South REGION
    unrecognized command:  / invalid command name
    r(199);

161 .
162 . lincom 1-2.region

     ( 1)  - 2.region = -1
```

	Coef.	Std. Err.	z	P>|z|	[95% Conf.	Interval]
(1)	.0003026	.0000304	9.94	0.000	.000243	.0003623

```
163 .
164 .
165 .
166 . //Comparison within North Region between Winter and Summer Season (Reference: WInter month)
    unrecognized command:  / invalid command name
    r(199);

167 .
168 . svy: logistic uc_cases_new  r1s
    (running logistic on estimation sample)

    Survey: Logistic regression

    Number of strata   =        36                Number of obs      =    861149
    Number of PSUs     =       244                Population size    = 4345308.2
                                                  Design df          =       208
                                                  F(   1,    208)    =      0.13
                                                  Prob > F           =    0.7150
```

uc_cases_new	Odds Ratio	Linearized Std. Err.	t	P>|t|	[95% Conf.	Interval]
r1s	**1.031173**	**.0865554**	**0.37**	**0.715**	**.8739057**	**1.216742**
_cons	**1756.775**	**306.4105**	**42.84**	**0.000**	**1245.615**	**2477.7**

Note: strata with single sampling unit centered at overall mean.

```
169 .
170 . //Comparison within South Region between Winter and Summer Season (Reference: WInter month)
```

unrecognized command: / invalid command name
r(199);

```
171 .
172 . svy: logistic uc_cases_new  r2s
(running logistic on estimation sample)

Survey: Logistic regression

Number of strata   =        26                Number of obs      =    857379
Number of PSUs     =       246                Population size    = 4012200.4
                                              Design df          =       220
                                              F(   1,    220)    =      0.21
                                              Prob > F           =    0.6451
```

uc_cases_new	Odds Ratio	Linearized Std. Err.	t	P>|t|	[95% Conf.	Interval]
r2s	**1.058395**	**.1302345**	**0.46**	**0.645**	**.830479**	**1.348859**
_cons	**3034.797**	**606.4071**	**40.13**	**0.000**	**2046.932**	**4499.413**

Note: strata with single sampling unit centered at overall mean.

```
173 .
174 .
175 .
176 . //************************************************************************************
```

unrecognized command: / invalid command name
r(199);

```
177 .
178 . // PART3. CD Cases only
```

unrecognized command: / invalid command name
r(199);

```
179 .
180 . ***************************************************************************************

181 .
182 . svy: logistic cd_cases_new i.region season_new
(running logistic on estimation sample)

Survey: Logistic regression
```

```
Number of strata   =        58                 Number of obs      =    1718528
Number of PSUs     =       490                 Population size    = 8357508.6
                                               Design df          =        432
                                               F(   2,     431)   =       5.66
                                               Prob > F           =     0.0037
```

cd_cases_new	Odds Ratio	Linearized Std. Err.	t	P>|t|	[95% Conf.	Interval]
2.region	1.440422	.1618528	3.25	0.001	1.154984	1.796402
season_new	.9777391	.0519688	-0.42	0.672	.8807503	1.085408
_cons	1007.584	116.0894	60.02	0.000	803.404	1263.655

```
Note: strata with single sampling unit centered at overall mean.

183 .
184 . svy: logistic cd_cases_new i.region
(running logistic on estimation sample)

Survey: Logistic regression

Number of strata   =        58                 Number of obs      =    4180307
Number of PSUs     =       543                 Population size    =   20203730
                                               Design df          =        485
                                               F(   1,     485)   =      22.21
                                               Prob > F           =     0.0000
```

cd_cases_new	Odds Ratio	Linearized Std. Err.	t	P>|t|	[95% Conf.	Interval]
2.region	1.536246	.139961	4.71	0.000	1.284451	1.837402
_cons	964.9174	67.57587	98.13	0.000	840.8702	1107.264

```
Note: strata with single sampling unit centered at overall mean.

185 .
186 . margins region

Adjusted predictions                             Number of obs    =    4180307
Model VCE    : Linearized

Expression   : Pr(cd_cases_new), predict()
```

	Margin	Delta-method Std. Err.	z	P>|z|	[95% Conf.	Interval]
region						
1	.9989647	.0000724	1.4e+04	0.000	.9988228	.9991067
2	.9993259	.0000395	2.5e+04	0.000	.9992485	.9994032

```
187 .
188 . margins region, post

    Adjusted predictions                              Number of obs   =    4180307
    Model VCE     : Linearized

    Expression    : Pr(cd_cases_new), predict()
```

	Margin (Delta-method)	Std. Err.	z	P>|z|	[95% Conf.	Interval]
region						
1	.9989647	.0000724	1.4e+04	0.000	.9988228	.9991067
2	.9993259	.0000395	2.5e+04	0.000	.9992485	.9994032

```
189 .
190 . // OVERALL INCIDENCE RATE CD only: North REGION
    unrecognized command:  / invalid command name
    r(199);

191 .
192 . lincom 1-1.region

     ( 1)  - 1bn.region = -1
```

	Coef.	Std. Err.	z	P>|z|	[95% Conf.	Interval]
(1)	.0010353	.0000724	14.29	0.000	.0008933	.0011772

```
193 .
194 . // OVERALL INCIDENCE RATE CD only: South REGION
    unrecognized command:  / invalid command name
    r(199);

195 .
196 . lincom 1-2.region

     ( 1)  - 2.region = -1
```

	Coef.	Std. Err.	z	P>|z|	[95% Conf.	Interval]
(1)	.0006741	.0000395	17.08	0.000	.0005968	.0007515

```
197 .
198 .
199 .
200 . //Comparison within North Region between Winter and Summer Season (Reference: WInter month)
```

unrecognized command: / invalid command name
r(199);

201 .
202 . svy: logistic cd_cases_new r1s
(running logistic on estimation sample)

Survey: Logistic regression

Number of strata =	**36**	Number of obs =	**861149**
Number of PSUs =	**244**	Population size =	**4345308.2**
		Design df =	**208**
		F(**1**, **208**) =	**0.31**
		Prob > F =	**0.5806**

cd_cases_new	Odds Ratio	Linearized Std. Err.	t	P>|t|	[95% Conf.	Interval]
r1s	**1.040318**	**.074316**	**0.55**	**0.581**	**.9036576**	**1.197646**
_cons	**917.9732**	**121.3946**	**51.59**	**0.000**	**707.3048**	**1191.388**

Note: strata with single sampling unit centered at overall mean.

203 .
204 . //Comparison within South Region between Winter and Summer Season (Reference: WInter month)
unrecognized command: / invalid command name
r(199);

205 .
206 . svy: logistic cd_cases_new r2s
(running logistic on estimation sample)

Survey: Logistic regression

Number of strata =	**26**	Number of obs =	**857379**
Number of PSUs =	**246**	Population size =	**4012200.4**
		Design df =	**220**
		F(**1**, **220**) =	**2.38**
		Prob > F =	**0.1246**

cd_cases_new	Odds Ratio	Linearized Std. Err.	t	P>|t|	[95% Conf.	Interval]
r2s	**.8874899**	**.0687193**	**-1.54**	**0.125**	**.761885**	**1.033802**
_cons	**1680.935**	**226.8481**	**55.03**	**0.000**	**1288.377**	**2193.103**

Note: strata with single sampling unit centered at overall mean.

207 . clear

208 . // YEAR 2003
unrecognized command: / invalid command name

```
    r(199);

209 . use "U:\RKunnavakkam\Adam_Stein\2003\UC_CD_2003_req.dta", clear

210 . //**********************************************************************
    unrecognized command:  / invalid command name
    r(199);

211 .
212 . // PART1. UC or CD cases
    unrecognized command:  / invalid command name
    r(199);

213 .
214 . //**********************************************************************
    unrecognized command:  / invalid command name
    r(199);

215 .
216 .
217 .
218 . svyset hospid [pweight=discwt], strata (nis_stratum) singleunit(centered)

          pweight: discwt
              VCE: linearized
      Single unit: centered
         Strata 1: nis_stratum
             SU 1: hospid
            FPC 1: <zero>

219 .
220 . svy: logistic uc_cd_new region season_new
    (running logistic on estimation sample)

    Survey: Logistic regression

    Number of strata   =        58                Number of obs      =   1766113
    Number of PSUs     =       483                Population size    = 8419553.4
                                                  Design df          =       425
                                                  F(   2,    424)    =      3.83
                                                  Prob > F           =    0.0226

    ------------------------------------------------------------------------------
                 |             Linearized
       uc_cd_new | Odds Ratio   Std. Err.      t    P>|t|     [95% Conf. Interval]
    -------------+----------------------------------------------------------------
          region |   1.432543   .2104967     2.45   0.015     1.073187    1.912228
      season_new |   .9463155   .0289884    -1.80   0.072     .8910184    1.005044
           _cons |   219.6049   56.81791    20.84   0.000     132.0637    365.1746
    ------------------------------------------------------------------------------
    Note: strata with single sampling unit centered at overall mean.

221 .
222 . svy: logistic uc_cd_new i.region
```

```
(running logistic on estimation sample)

Survey: Logistic regression

Number of strata   =        58                  Number of obs      =    4215803
Number of PSUs     =       542                  Population size    =   19903380
                                                Design df          =        484
                                                F(   1,    484)    =       5.92
                                                Prob > F           =     0.0153
```

uc_cd_new	Odds Ratio	Linearized Std. Err.	t	P>|t|	[95% Conf.	Interval]
2.region	1.391106	.1886805	2.43	0.015	1.065662	1.815938
_cons	290.7338	35.4818	46.48	0.000	228.7455	369.5205

Note: strata with single sampling unit centered at overall mean.

```
223 .
224 . margins region

Adjusted predictions                              Number of obs   =    4215803
Model VCE    : Linearized

Expression   : Pr(uc_cd_new), predict()
```

	Margin	Delta-method Std. Err.	z	P>|z|	[95% Conf.	Interval]
region						
1	.9965722	.0004169	2390.43	0.000	.9957551	.9973893
2	.9975336	.0001456	6849.57	0.000	.9972481	.997819

```
225 .
226 . margins region, post

Adjusted predictions                              Number of obs   =    4215803
Model VCE    : Linearized

Expression   : Pr(uc_cd_new), predict()
```

	Margin	Delta-method Std. Err.	z	P>|z|	[95% Conf.	Interval]
region						
1	.9965722	.0004169	2390.43	0.000	.9957551	.9973893
2	.9975336	.0001456	6849.57	0.000	.9972481	.997819

```
227 .
```

```
228 . // OVERALL INCIDENCE RATE UC or CD cases : North REGION
    unrecognized command:  / invalid command name
    r(199);

229 .
230 . lincom 1-1.region

     ( 1)  - 1bn.region = -1
```

	Coef.	Std. Err.	z	P>|z|	[95% Conf.	Interval]
(1)	.0034278	.0004169	8.22	0.000	.0026107	.0042449

```
231 .
232 . // OVERALL INCIDENCE RATE UC or CD cases : South REGION
    unrecognized command:  / invalid command name
    r(199);

233 .
234 . lincom 1-2.region

     ( 1)  - 2.region = -1
```

	Coef.	Std. Err.	z	P>|z|	[95% Conf.	Interval]
(1)	.0024664	.0001456	16.94	0.000	.002181	.0027519

```
235 .
236 . // This gives results for within region comparing season1 and season 2
    unrecognized command:  / invalid command name
    r(199);

237 .
238 . //Comparison within North Region between Winter and Summer Season (Reference: WInter month)
    unrecognized command:  / invalid command name
    r(199);

239 .
240 . svy: logistic uc_cd_new  r1s
    (running logistic on estimation sample)

    Survey: Logistic regression

    Number of strata   =        37                  Number of obs      =    799489
    Number of PSUs     =       221                  Population size    = 3956949.7
                                                    Design df          =       184
                                                    F(   1,    184)    =      3.37
                                                    Prob > F           =    0.0680
```

```
                                Linearized
    uc_cd_new   Odds Ratio   Std. Err.      t    P>|t|     [95% Conf. Interval]
-------------+----------------------------------------------------------------
         r1s     .9320047    .0357488    -1.84   0.068     .8640772    1.005272
       _cons     321.9499    44.58493    41.70   0.000     244.9797    423.1035
------------------------------------------------------------------------------
Note: strata with single sampling unit centered at overall mean.

241 .
242 . //Comparison within South Region between Winter and Summer Season (Reference: WInter month
    unrecognized command:  / invalid command name
    r(199);

243 .
244 . svy: logistic uc_cd_new   r2s
    (running logistic on estimation sample)

    Survey: Logistic regression

    Number of strata   =        32                Number of obs      =    966624
    Number of PSUs     =       262                Population size    = 4462603.6
                                                  Design df          =       230
                                                  F(   1,    230)    =      0.54
                                                  Prob > F           =    0.4647

------------------------------------------------------------------------------
                                Linearized
    uc_cd_new   Odds Ratio   Std. Err.      t    P>|t|     [95% Conf. Interval]
-------------+----------------------------------------------------------------
         r2s     .9647604     .047256    -0.73   0.465     .8760024    1.062512
       _cons     437.7266    57.73487    46.11   0.000       337.55    567.6333
------------------------------------------------------------------------------
Note: strata with single sampling unit centered at overall mean.

245 .
246 .
247 .
248 . //*****************************************************************************************
    unrecognized command:  / invalid command name
    r(199);

249 .
250 . // PART2. UC cases only
    unrecognized command:  / invalid command name
    r(199);

251 .
252 . //****************************************************************************************
    unrecognized command:  / invalid command name
    r(199);

253 .
254 . svy: logistic uc_cases_new i.region season_new
    (running logistic on estimation sample)
```

Survey: Logistic regression

Number of strata	= **58**		Number of obs	= **1766113**
Number of PSUs	= **483**		Population size	= **8419553.4**
			Design df	= **425**
			F(2, 424)	= **3.01**
			Prob > F	= **0.0505**

uc_cases_new	Odds Ratio	Linearized Std. Err.	t	P>|t|	[95% Conf.	Interval]
2.region	**1.43177**	**.2241606**	**2.29**	**0.022**	**1.052512**	**1.947689**
season_new	**.9297857**	**.0450359**	**-1.50**	**0.134**	**.8453481**	**1.022657**
_cons	**809.8794**	**117.584**	**46.13**	**0.000**	**608.813**	**1077.35**

Note: strata with single sampling unit centered at overall mean.

255 .
256 . margins region

Predictive margins   Number of obs = **1766113**
Model VCE : **Linearized**

Expression : **Pr(uc_cases_new), predict()**

	Margin	Delta-method Std. Err.	z	P>|z|	[95% Conf.	Interval]
region						
1	**.9986238**	**.0001902**	**5249.52**	**0.000**	**.998251**	**.9989966**
2	**.9990384**	**.0000703**	**1.4e+04**	**0.000**	**.9989006**	**.9991762**

257 .
258 . margins region, post

Predictive margins   Number of obs = **1766113**
Model VCE : **Linearized**

Expression : **Pr(uc_cases_new), predict()**

	Margin	Delta-method Std. Err.	z	P>|z|	[95% Conf.	Interval]
region						
1	**.9986238**	**.0001902**	**5249.52**	**0.000**	**.998251**	**.9989966**
2	**.9990384**	**.0000703**	**1.4e+04**	**0.000**	**.9989006**	**.9991762**

259 .

```
260 . // OVERALL INCIDENCE RATE UC only: North REGION
    unrecognized command:  / invalid command name
    r(199);

261 .
262 . lincom 1-1.region

     ( 1)  - 1bn.region = -1
```

	Coef.	Std. Err.	z	P>|z|	[95% Conf.	Interval]
(1)	.0013762	.0001902	7.23	0.000	.0010034	.001749

```
263 .
264 . // OVERALL INCIDENCE RATE UC only : South REGION
    unrecognized command:  / invalid command name
    r(199);

265 .
266 . lincom 1-2.region

     ( 1)  - 2.region = -1
```

	Coef.	Std. Err.	z	P>|z|	[95% Conf.	Interval]
(1)	.0009616	.0000703	13.67	0.000	.0008238	.0010994

```
267 .
268 .
269 .
270 . //Comparison within North Region between Winter and Summer Season (Reference: WInter month)
    unrecognized command:  / invalid command name
    r(199);

271 .
272 . svy: logistic uc_cases_new  r1s
    (running logistic on estimation sample)

    Survey: Logistic regression

    Number of strata   =        37                 Number of obs      =    799489
    Number of PSUs     =       221                 Population size    = 3956949.7
                                                   Design df          =       184
                                                   F(   1,    184)    =      2.55
                                                   Prob > F           =    0.1118
```

uc_cases_new	Odds Ratio	Linearized Std. Err.	t	P>|t|	[95% Conf.	Interval]

```
         r1s |    .906655   .0555981    -1.60   0.112      .803339    1.023258
       _cons |   841.5475   127.9024    44.32   0.000      623.523    1135.808
------------------------------------------------------------------------------
Note: strata with single sampling unit centered at overall mean.
```

273 .
274 . //Comparison within South Region between Winter and Summer Season (Reference: WInter month)
unrecognized command: / invalid command name
r(199);

275 .
276 . svy: logistic uc_cases_new r2s
(running logistic on estimation sample)

Survey: Logistic regression

```
Number of strata   =        32                  Number of obs      =    966624
Number of PSUs     =       262                  Population size    = 4462603.6
                                                Design df          =       230
                                                F(   1,    230)    =      0.29
                                                Prob > F           =    0.5938
```

uc_cases_new	Odds Ratio	Linearized Std. Err.	t	P>|t|	[95% Conf.	Interval]
r2s	.9599407	.0734731	-0.53	0.594	.8255616	1.116193
_cons	1104.921	175.63	44.09	0.000	807.8201	1511.289

Note: strata with single sampling unit centered at overall mean.

277 .
278 .
279 .
280 . //***
unrecognized command: / invalid command name
r(199);

281 .
282 . // PART3. CD Cases only
unrecognized command: / invalid command name
r(199);

283 .
284 . **

285 .
286 . svy: logistic cd_cases_new i.region season_new
(running logistic on estimation sample)

Survey: Logistic regression

```
Number of strata   =        58                  Number of obs      =   1766113
Number of PSUs     =       483                  Population size    = 8419553.4
```

```
                                        Design df          =        425
                                        F(   2,    424)    =       3.67
                                        Prob > F           =     0.0262
```

cd_cases_new	Odds Ratio	Linearized Std. Err.	t	P>|t|	[95% Conf.	Interval]
2.region	**1.437211**	**.210598**	**2.48**	**0.014**	**1.077546**	**1.916926**
season_new	**.954554**	**.0395577**	**-1.12**	**0.262**	**.8798832**	**1.035562**
_cons	**481.2049**	**68.80261**	**43.20**	**0.000**	**363.3103**	**637.3563**

Note: strata with single sampling unit centered at overall mean.

```
287 .
288 . svy: logistic cd_cases_new i.region
    (running logistic on estimation sample)

    Survey: Logistic regression

    Number of strata   =        58              Number of obs      =    4215803
    Number of PSUs     =       542              Population size    =   19903380
                                                Design df          =        484
                                                F(   1,    484)    =       5.97
                                                Prob > F           =     0.0149
```

cd_cases_new	Odds Ratio	Linearized Std. Err.	t	P>|t|	[95% Conf.	Interval]
2.region	**1.390373**	**.187573**	**2.44**	**0.015**	**1.066619**	**1.812398**
_cons	**447.3038**	**53.4034**	**51.12**	**0.000**	**353.7719**	**565.5643**

Note: strata with single sampling unit centered at overall mean.

```
289 .
290 . margins region

    Adjusted predictions                          Number of obs   =    4215803
    Model VCE    : Linearized

    Expression   : Pr(cd_cases_new), predict()
```

	Margin	Delta-method Std. Err.	z	P>|z|	[95% Conf.	Interval]
region						
1	**.9977694**	**.0002657**	**3754.97**	**0.000**	**.9972486**	**.9982902**
2	**.9983947**	**.0001006**	**9923.55**	**0.000**	**.9981975**	**.9985918**

```
291 .
292 . margins region, post
```

```
Adjusted predictions                              Number of obs   =    4215803
Model VCE    : Linearized

Expression   : Pr(cd_cases_new), predict()
```

	Delta-method					
	Margin	Std. Err.	z	P>|z|	[95% Conf.	Interval]
region						
1	.9977694	.0002657	3754.97	0.000	.9972486	.9982902
2	.9983947	.0001006	9923.55	0.000	.9981975	.9985918

```
293 .
294 . // OVERALL INCIDENCE RATE CD only: North REGION
    unrecognized command:  / invalid command name
    r(199);

295 .
296 . lincom 1-1.region

     ( 1)  - 1bn.region = -1
```

	Coef.	Std. Err.	z	P>|z|	[95% Conf.	Interval]
(1)	.0022306	.0002657	8.39	0.000	.0017098	.0027514

```
297 .
298 . // OVERALL INCIDENCE RATE CD only: South REGION
    unrecognized command:  / invalid command name
    r(199);

299 .
300 . lincom 1-2.region

     ( 1)  - 2.region = -1
```

	Coef.	Std. Err.	z	P>|z|	[95% Conf.	Interval]
(1)	.0016053	.0001006	15.96	0.000	.0014082	.0018025

```
301 .
302 .
303 .
304 . //Comparison within North Region between Winter and Summer Season (Reference: WInter month)
    unrecognized command:  / invalid command name
    r(199);
```

```
305 .
306 . svy: logistic cd_cases_new  r1s
    (running logistic on estimation sample)

    Survey: Logistic regression

    Number of strata   =        37                  Number of obs      =    799489
    Number of PSUs     =       221                  Population size    = 3956949.7
                                                    Design df          =       184
                                                    F(   1,    184)    =      0.93
                                                    Prob > F           =    0.3353
```

cd_cases_new	Odds Ratio	Linearized Std. Err.	t	P>|t|	[95% Conf.	Interval]
r1s	**.9472292**	**.0531599**	**-0.97**	**0.335**	**.8479458**	**1.058137**
_cons	**486.8493**	**78.18423**	**38.53**	**0.000**	**354.6444**	**668.3377**

```
    Note: strata with single sampling unit centered at overall mean.

307 .
308 . //Comparison within South Region between Winter and Summer Season (Reference: WInter month)
    unrecognized command:  / invalid command name
    r(199);

309 .
310 . svy: logistic cd_cases_new  r2s
    (running logistic on estimation sample)

    Survey: Logistic regression

    Number of strata   =        32                  Number of obs      =    966624
    Number of PSUs     =       262                  Population size    = 4462603.6
                                                    Design df          =       230
                                                    F(   1,    230)    =      0.35
                                                    Prob > F           =    0.5540
```

cd_cases_new	Odds Ratio	Linearized Std. Err.	t	P>|t|	[95% Conf.	Interval]
r2s	**.9639547**	**.0597144**	**-0.59**	**0.554**	**.8531945**	**1.089094**
_cons	**681.4443**	**97.92127**	**45.40**	**0.000**	**513.4149**	**904.4659**

```
    Note: strata with single sampling unit centered at overall mean.

311 . clear

312 . use "U:\RKunnavakkam\Adam_Stein\2004\UC_CD_2004_req.dta", clear

313 . //YEAR 2004
    unrecognized command:  / invalid command name
    r(199);
```

```
314 . //*********************************************************************
    unrecognized command:  / invalid command name
    r(199);

315 .
316 . // PART1. UC or CD cases
    unrecognized command:  / invalid command name
    r(199);

317 .
318 . //*********************************************************************
    unrecognized command:  / invalid command name
    r(199);

319 .
320 .
321 .
322 . svyset hospid [pweight=discwt], strata (nis_stratum) singleunit(centered)

          pweight: discwt
              VCE: linearized
      Single unit: centered
         Strata 1: nis_stratum
             SU 1: hospid
            FPC 1: <zero>

323 .
324 . svy: logistic uc_cd_new region season_new
    (running logistic on estimation sample)

    Survey: Logistic regression

    Number of strata   =        58                 Number of obs      =   2023240
    Number of PSUs     =       541                 Population size    = 9664823.3
                                                   Design df          =       483
                                                   F(   2,    482)    =     10.52
                                                   Prob > F           =    0.0000
```

uc_cd_new	Odds Ratio	Linearized Std. Err.	t	P>|t|	[95% Conf.	Interval]
region	1.718777	.2148208	4.33	0.000	1.344513	2.197222
season_new	.958824	.0242077	-1.67	0.096	.9124191	1.007589
_cons	161.9594	38.17299	21.58	0.000	101.9244	257.3558

```
    Note: strata with single sampling unit centered at overall mean.

325 .
326 . svy: logistic uc_cd_new i.region
    (running logistic on estimation sample)

    Survey: Logistic regression
```

```
Number of strata   =        58                Number of obs      =    4738069
Number of PSUs     =       597                Population size    =   22482509
                                              Design df          =        539
                                              F(   1,    539)    =      11.76
                                              Prob > F           =     0.0007
```

uc_cd_new	Odds Ratio	Linearized Std. Err.	t	P>|t|	[95% Conf.	Interval]
2.region	**1.520892**	**.1859981**	**3.43**	**0.001**	**1.196096**	**1.933885**
_cons	**263.2022**	**29.91894**	**49.03**	**0.000**	**210.5296**	**329.0529**

Note: strata with single sampling unit centered at overall mean.

```
327 .
328 . margins region
```

```
Adjusted predictions                            Number of obs     =    4738069
Model VCE    : Linearized

Expression   : Pr(uc_cd_new), predict()
```

	Margin	Delta-method Std. Err.	z	P>|z|	[95% Conf.	Interval]
region						
1	**.996215**	**.0004286**	**2324.23**	**0.000**	**.9953749**	**.9970551**
2	**.9975081**	**.000111**	**8989.79**	**0.000**	**.9972906**	**.9977256**

```
329 .
330 . margins region, post
```

```
Adjusted predictions                            Number of obs     =    4738069
Model VCE    : Linearized

Expression   : Pr(uc_cd_new), predict()
```

	Margin	Delta-method Std. Err.	z	P>|z|	[95% Conf.	Interval]
region						
1	**.996215**	**.0004286**	**2324.23**	**0.000**	**.9953749**	**.9970551**
2	**.9975081**	**.000111**	**8989.79**	**0.000**	**.9972906**	**.9977256**

```
331 .
332 . // OVERALL INCIDENCE RATE UC or CD cases : North REGION
unrecognized command:  / invalid command name
r(199);
```

```
333 .
334 . lincom 1-1.region

 ( 1)  - 1bn.region = -1

------------------------------------------------------------------------------
             |      Coef.   Std. Err.      z    P>|z|     [95% Conf. Interval]
-------------+----------------------------------------------------------------
         (1) |    .003785   .0004286     8.83   0.000     .0029449    .0046251
------------------------------------------------------------------------------

335 .
336 . // OVERALL INCIDENCE RATE UC or CD cases : South REGION
unrecognized command:  / invalid command name
r(199);

337 .
338 . lincom 1-2.region

 ( 1)  - 2.region = -1

------------------------------------------------------------------------------
             |      Coef.   Std. Err.      z    P>|z|     [95% Conf. Interval]
-------------+----------------------------------------------------------------
         (1) |   .0024919    .000111    22.46   0.000     .0022744    .0027094
------------------------------------------------------------------------------

339 .
340 . // This gives results for within region comparing season1 and season 2
unrecognized command:  / invalid command name
r(199);

341 .
342 . //Comparison within North Region between Winter and Summer Season (Reference: WInter month)
unrecognized command:  / invalid command name
r(199);

343 .
344 . svy: logistic uc_cd_new  r1s
(running logistic on estimation sample)

Survey: Logistic regression

Number of strata   =        36                  Number of obs      =    988507
Number of PSUs     =       258                  Population size    = 4834112.3
                                                Design df          =       222
                                                F(   1,    222)    =      0.25
                                                Prob > F           =    0.6199

------------------------------------------------------------------------------
             |             Linearized
   uc_cd_new | Odds Ratio   Std. Err.      t    P>|t|     [95% Conf. Interval]
-------------+----------------------------------------------------------------
```

```
         r1s |   .9852911   .0293915    -0.50   0.620     .9290386     1.04495
       _cons |   267.1501   31.07259    48.04   0.000     212.4263    335.9715
------------------------------------------------------------------------------
Note: strata with single sampling unit centered at overall mean.
```

345 .

346 . //Comparison within South Region between Winter and Summer Season (Reference: WInter month

unrecognized command: / invalid command name

r(199);

347 .

348 . svy: logistic uc_cd_new   r2s

```
(running logistic on estimation sample)

Survey: Logistic regression

Number of strata   =        33                  Number of obs      =   1034733
Number of PSUs     =       283                  Population size    =   4830711
                                                Design df          =       250
                                                F(   1,    250)    =      3.64
                                                Prob > F           =    0.0576

------------------------------------------------------------------------------
             |             Linearized
   uc_cd_new | Odds Ratio   Std. Err.      t    P>|t|     [95% Conf. Interval]
-------------+----------------------------------------------------------------
         r2s |   .9150636   .0425825    -1.91   0.058      .834926    1.002893
       _cons |   513.5339   47.59459    67.34   0.000      427.854    616.3717
------------------------------------------------------------------------------
Note: strata with single sampling unit centered at overall mean.
```

349 .

350 .

351 .

352 . //***

unrecognized command: / invalid command name

r(199);

353 .

354 . // PART2. UC cases only

unrecognized command: / invalid command name

r(199);

355 .

356 . //**

unrecognized command: / invalid command name

r(199);

357 .

358 . svy: logistic uc_cases_new i.region season_new

(running logistic on estimation sample)

Survey: Logistic regression

```
Number of strata   =        58                 Number of obs      =   2023240
Number of PSUs     =       541                 Population size    = 9664823.3
                                               Design df          =       483
                                               F(   2,    482)    =      8.85
                                               Prob > F           =    0.0002
```

uc_cases_new	Odds Ratio	Linearized Std. Err.	t	P>|t|	[95% Conf.	Interval]
2.region	1.726057	.2345581	4.02	0.000	1.321577	2.25433
season_new	.9696014	.0402838	-0.74	0.458	.8935928	1.052075
_cons	718.712	102.3124	46.20	0.000	543.3473	950.6755

Note: strata with single sampling unit centered at overall mean.

```
359 .
360 . margins region
```

```
Predictive margins                                Number of obs     =    2023240
Model VCE    : Linearized

Expression   : Pr(uc_cases_new), predict()
```

	Margin	Delta-method Std. Err.	z	P>|z|	[95% Conf.	Interval]
region						
1	.9985446	.0001749	5708.30	0.000	.9982017	.9988874
2	.9991563	.0000533	1.9e+04	0.000	.9990518	.9992608

```
361 .
362 . margins region, post
```

```
Predictive margins                                Number of obs     =    2023240
Model VCE    : Linearized

Expression   : Pr(uc_cases_new), predict()
```

	Margin	Delta-method Std. Err.	z	P>|z|	[95% Conf.	Interval]
region						
1	.9985446	.0001749	5708.30	0.000	.9982017	.9988874
2	.9991563	.0000533	1.9e+04	0.000	.9990518	.9992608

```
363 .
364 . // OVERALL INCIDENCE RATE UC only: North REGION
unrecognized command:  / invalid command name
r(199);
```

```
365 .
366 . lincom 1-1.region

 ( 1)  - 1bn.region = -1

------------------------------------------------------------------------------
             |      Coef.   Std. Err.      z    P>|z|     [95% Conf. Interval]
-------------+----------------------------------------------------------------
         (1) |   .0014554   .0001749     8.32   0.000     .0011126    .0017983
------------------------------------------------------------------------------

367 .
368 . // OVERALL INCIDENCE RATE UC only : South REGION
    unrecognized command:  / invalid command name
    r(199);

369 .
370 . lincom 1-2.region

 ( 1)  - 2.region = -1

------------------------------------------------------------------------------
             |      Coef.   Std. Err.      z    P>|z|     [95% Conf. Interval]
-------------+----------------------------------------------------------------
         (1) |   .0008437   .0000533    15.82   0.000     .0007392    .0009482
------------------------------------------------------------------------------

371 .
372 .
373 .
374 . //Comparison within North Region between Winter and Summer Season (Reference: WInter month)
    unrecognized command:  / invalid command name
    r(199);

375 .
376 . svy: logistic uc_cases_new  r1s
    (running logistic on estimation sample)

    Survey: Logistic regression

    Number of strata   =        36                  Number of obs      =    988507
    Number of PSUs     =       258                  Population size    = 4834112.3
                                                    Design df          =       222
                                                    F(   1,    222)    =      1.64
                                                    Prob > F           =    0.2018

------------------------------------------------------------------------------
             |             Linearized
uc_cases_new | Odds Ratio   Std. Err.      t    P>|t|     [95% Conf. Interval]
-------------+----------------------------------------------------------------
         r1s |   1.059809   .0480853     1.28   0.202     .9691601    1.158937
       _cons |   628.8918   83.97379    48.26   0.000     483.3869    818.1954
------------------------------------------------------------------------------
```

Note: strata with single sampling unit centered at overall mean.

377 .
378 . //Comparison within South Region between Winter and Summer Season (Reference: WInter month)
unrecognized command: / invalid command name
r(199);

379 .
380 . svy: logistic uc_cases_new r2s
(running logistic on estimation sample)

Survey: Logistic regression

Number of strata =	**33**	Number of obs =	**1034733**
Number of PSUs =	**283**	Population size =	**4830711**
		Design df =	**250**
		F(**1**, **250**) =	**5.03**
		Prob > F =	**0.0258**

uc_cases_new	Odds Ratio	Linearized Std. Err.	t	P>|t|	[95% Conf.	Interval]
r2s	**.831268**	**.0685209**	**-2.24**	**0.026**	**.7067011**	**.9777917**
_cons	**1568.779**	**230.0931**	**50.17**	**0.000**	**1175.192**	**2094.185**

Note: strata with single sampling unit centered at overall mean.

381 .
382 .
383 .
384 . //**
unrecognized command: / invalid command name
r(199);

385 .
386 . // PART3. CD Cases only
unrecognized command: / invalid command name
r(199);

387 .
388 . ***

389 .
390 . svy: logistic cd_cases_new i.region season_new
(running logistic on estimation sample)

Survey: Logistic regression

Number of strata =	**58**	Number of obs =	**2023240**
Number of PSUs =	**541**	Population size =	**9664823.3**
		Design df =	**483**
		F(**2**, **482**) =	**10.13**
		Prob > F =	**0.0000**

cd_cases_new	Odds Ratio	Linearized Std. Err.	t	P>|t|	[95% Conf.	Interval]
2.region	**1.739291**	**.2193824**	**4.39**	**0.000**	**1.357493**	**2.22847**
season_new	**.9568585**	**.0315453**	**-1.34**	**0.182**	**.8968404**	**1.020893**
_cons	**422.7595**	**51.31455**	**49.82**	**0.000**	**333.0542**	**536.6261**

Note: strata with single sampling unit centered at overall mean.

```
391 .
392 . svy: logistic cd_cases_new i.region
(running logistic on estimation sample)

Survey: Logistic regression

Number of strata   =        58                  Number of obs      =   4738069
Number of PSUs     =       597                  Population size    =  22482509
                                                Design df          =       539
                                                F(   1,    539)    =     12.64
                                                Prob > F           =    0.0004
```

cd_cases_new	Odds Ratio	Linearized Std. Err.	t	P>|t|	[95% Conf.	Interval]
2.region	**1.550219**	**.1911311**	**3.56**	**0.000**	**1.216773**	**1.975042**
_cons	**400.8016**	**46.18203**	**52.02**	**0.000**	**319.6169**	**502.6078**

Note: strata with single sampling unit centered at overall mean.

```
393 .
394 . margins region

Adjusted predictions                              Number of obs    =    4738069
Model VCE    : Linearized

Expression   : Pr(cd_cases_new), predict()
```

	Margin	Delta-method Std. Err.	z	P>|z|	[95% Conf.	Interval]
region						
1	**.9975112**	**.0002861**	**3487.13**	**0.000**	**.9969506**	**.9980719**
2	**.9983931**	**.0000692**	**1.4e+04**	**0.000**	**.9982575**	**.9985288**

```
395 .
396 . margins region, post

Adjusted predictions                              Number of obs    =    4738069
Model VCE    : Linearized
```

```
Expression   : Pr(cd_cases_new), predict()
```

	Margin (Delta-method)	Std. Err. (Delta-method)	z	P>|z|	[95% Conf.	Interval]
region						
1	.9975112	.0002861	3487.13	0.000	.9969506	.9980719
2	.9983931	.0000692	1.4e+04	0.000	.9982575	.9985288

```
397 .
398 . // OVERALL INCIDENCE RATE CD only: North REGION
unrecognized command:  / invalid command name
r(199);

399 .
400 . lincom 1-1.region

 ( 1)  - 1bn.region = -1
```

	Coef.	Std. Err.	z	P>|z|	[95% Conf.	Interval]
(1)	.0024888	.0002861	8.70	0.000	.0019281	.0030494

```
401 .
402 . // OVERALL INCIDENCE RATE CD only: South REGION
unrecognized command:  / invalid command name
r(199);

403 .
404 . lincom 1-2.region

 ( 1)  - 2.region = -1
```

	Coef.	Std. Err.	z	P>|z|	[95% Conf.	Interval]
(1)	.0016069	.0000692	23.22	0.000	.0014712	.0017425

```
405 .
406 .
407 .
408 . //Comparison within North Region between Winter and Summer Season (Reference: WInter month)
unrecognized command:  / invalid command name
r(199);

409 .
410 . svy: logistic cd_cases_new  r1s
(running logistic on estimation sample)
```

```
Survey: Logistic regression

Number of strata   =        36                  Number of obs      =    988507
Number of PSUs     =       258                  Population size    = 4834112.3
                                                Design df          =       222
                                                F(   1,    222)    =      0.74
                                                Prob > F           =    0.3910

------------------------------------------------------------------------------
             |             Linearized
cd_cases_new | Odds Ratio   Std. Err.      t    P>|t|     [95% Conf. Interval]
-------------+----------------------------------------------------------------
         r1s |     .96452    .040536    -0.86   0.391     .8878541    1.047806
       _cons |   417.6855   51.86542    48.60   0.000     327.0193    533.4888
------------------------------------------------------------------------------
Note: strata with single sampling unit centered at overall mean.

411 .
412 . //Comparison within South Region between Winter and Summer Season (Reference: WInter month)
    unrecognized command:  / invalid command name
    r(199);

413 .
414 . svy: logistic cd_cases_new  r2s
    (running logistic on estimation sample)

Survey: Logistic regression

Number of strata   =        33                  Number of obs      =   1034733
Number of PSUs     =       283                  Population size    =   4830711
                                                Design df          =       250
                                                F(   1,    250)    =      1.20
                                                Prob > F           =    0.2740

------------------------------------------------------------------------------
             |             Linearized
cd_cases_new | Odds Ratio   Std. Err.      t    P>|t|     [95% Conf. Interval]
-------------+----------------------------------------------------------------
         r2s |   .9436972   .0498788    -1.10   0.274     .8504011    1.047229
       _cons |   750.8509    76.5634    64.93   0.000     614.2362    917.8505
------------------------------------------------------------------------------
Note: strata with single sampling unit centered at overall mean.

415 . clear

416 . use "U:\RKunnavakkam\Adam_Stein\2005\UC_CD_2005_req.dta", clear

417 . //**********************************************************************
    unrecognized command:  / invalid command name
    r(199);

418 .
419 . // PART1. UC or CD cases
```

```
    unrecognized command:  / invalid command name
    r(199);

420 .
421 . //********************************************************************
    unrecognized command:  / invalid command name
    r(199);

422 .
423 .
424 .
425 . svyset hospid [pweight=discwt], strata (nis_stratum) singleunit(centered)

          pweight: discwt
              VCE: linearized
      Single unit: centered
         Strata 1: nis_stratum
             SU 1: hospid
            FPC 1: <zero>

426 .
427 . svy: logistic uc_cd_new region season_new
    (running logistic on estimation sample)

    Survey: Logistic regression

    Number of strata   =        59                 Number of obs      =   2100195
    Number of PSUs     =       607                 Population size    =  10289325
                                                   Design df          =       548
                                                   F(   2,    547)    =     13.73
                                                   Prob > F           =    0.0000

    ------------------------------------------------------------------------------
                 |             Linearized
       uc_cd_new | Odds Ratio   Std. Err.      t    P>|t|     [95% Conf. Interval]
    -------------+----------------------------------------------------------------
          region |   1.621051   .1494263     5.24   0.000     1.352572    1.942822
      season_new |   1.034598   .0463113     0.76   0.448     .9475137    1.129687
           _cons |   378.9618   57.42823    39.18   0.000     281.3961    510.3555
    ------------------------------------------------------------------------------
    Note: strata with single sampling unit centered at overall mean.

428 .
429 . svy: logistic uc_cd_new i.region
    (running logistic on estimation sample)

    Survey: Logistic regression

    Number of strata   =        59                 Number of obs      =   4819620
    Number of PSUs     =       658                 Population size    =  23552265
                                                   Design df          =       599
                                                   F(   1,    599)    =     32.25
                                                   Prob > F           =    0.0000
```

uc_cd_new	Odds Ratio	Linearized Std. Err.	t	P>|t|	[95% Conf.	Interval]
2.region	**1.572614**	**.1253725**	**5.68**	**0.000**	**1.344699**	**1.83916**
_cons	**656.9747**	**34.88432**	**122.18**	**0.000**	**591.9154**	**729.1848**

Note: strata with single sampling unit centered at overall mean.

430 .
431 . margins region

Adjusted predictions Number of obs = **4819620**
Model VCE : **Linearized**

Expression : **Pr(uc_cd_new), predict()**

	Margin	Delta-method Std. Err.	z	P>|z|	[95% Conf.	Interval]
region						
1	**.9984802**	**.0000806**	**1.2e+04**	**0.000**	**.9983223**	**.9986381**
2	**.999033**	**.0000575**	**1.7e+04**	**0.000**	**.9989203**	**.9991458**

432 .
433 . margins region, post

Adjusted predictions Number of obs = **4819620**
Model VCE : **Linearized**

Expression : **Pr(uc_cd_new), predict()**

	Margin	Delta-method Std. Err.	z	P>|z|	[95% Conf.	Interval]
region						
1	**.9984802**	**.0000806**	**1.2e+04**	**0.000**	**.9983223**	**.9986381**
2	**.999033**	**.0000575**	**1.7e+04**	**0.000**	**.9989203**	**.9991458**

434 .
435 . / OVERALL INCIDENCE RATE UC or CD cases : North REGION
unrecognized command: / invalid command name
r(199);

436 . lincom 1-1.region

(1) **- 1bn.region = -1**

	Coef.	Std. Err.	z	P>|z|	[95% Conf.	Interval]

(1)	**.0015198**	**.0000806**	**18.86**	**0.000**	**.0013619**	**.0016777**

```
437 . // OVERALL INCIDENCE RATE UC or CD cases : South REGION
    unrecognized command:  / invalid command name
    r(199);

438 . lincom 1-2.region

     ( 1)  - 2.region = -1
```

	Coef.	Std. Err.	z	P>|z|	[95% Conf.	Interval]
(1)	**.000967**	**.0000575**	**16.81**	**0.000**	**.0008542**	**.0010797**

```
439 . // This gives results for within region comparing season1 and season 2
    unrecognized command:  / invalid command name
    r(199);

440 . //Comparison within North Region between Winter and Summer Season (Reference: WInter month)
    unrecognized command:  / invalid command name
    r(199);

441 . svy: logistic uc_cd_new  r1s
    (running logistic on estimation sample)

    Survey: Logistic regression

    Number of strata   =        37                Number of obs      =    980627
    Number of PSUs     =       273                Population size    = 4877690.8
                                                  Design df          =       236
                                                  F(   1,    236)    =      1.88
                                                  Prob > F           =    0.1719
```

uc_cd_new	Odds Ratio	Linearized Std. Err.	t	P>|t|	[95% Conf.	Interval]
r1s	**1.085044**	**.0646302**	**1.37**	**0.172**	**.9649047**	**1.220141**
_cons	**572.2046**	**60.11215**	**60.44**	**0.000**	**465.2309**	**703.7753**

```
    Note: strata with single sampling unit centered at overall mean.

442 . //Comparison within South Region between Winter and Summer Season (Reference: WInter month
    unrecognized command:  / invalid command name
    r(199);

443 . svy: logistic uc_cd_new   r2s
    (running logistic on estimation sample)

    Survey: Logistic regression
```

```
Number of strata   =        35               Number of obs      =   1119568
Number of PSUs     =       334               Population size    = 5411634.2
                                             Design df          =       299
                                             F(   1,    299)    =      0.30
                                             Prob > F           =    0.5840
```

uc_cd_new	Odds Ratio	Linearized Std. Err.	t	P>|t|	[95% Conf.	Interval]
r2s	**.9651923**	**.0623744**	**-0.55**	**0.584**	**.8499285**	**1.096088**
_cons	**1104.906**	**136.5129**	**56.72**	**0.000**	**866.424**	**1409.029**

Note: strata with single sampling unit centered at overall mean.

```
444 .
445 . //*******************************************************************************************
    unrecognized command:  / invalid command name
    r(199);

446 . // PART2. UC cases only
    unrecognized command:  / invalid command name
    r(199);

447 . //******************************************************************************************
    unrecognized command:  / invalid command name
    r(199);

448 . svy: logistic uc_cases_new i.region season_new
    (running logistic on estimation sample)

    Survey: Logistic regression

    Number of strata   =        59               Number of obs      =   2100195
    Number of PSUs     =       607               Population size    =  10289325
                                                 Design df          =       548
                                                 F(   2,    547)    =      8.39
                                                 Prob > F           =    0.0003
```

uc_cases_new	Odds Ratio	Linearized Std. Err.	t	P>|t|	[95% Conf.	Interval]
2.region	**1.654095**	**.2064029**	**4.03**	**0.000**	**1.294523**	**2.113543**
season_new	**1.066629**	**.0720063**	**0.96**	**0.340**	**.934164**	**1.217878**
_cons	**1654.041**	**206.9905**	**59.22**	**0.000**	**1293.567**	**2114.966**

Note: strata with single sampling unit centered at overall mean.

```
449 . margins region

    Predictive margins                               Number of obs     =    2100195
    Model VCE    : Linearized
```

Expression : **Pr(uc_cases_new), predict()**

	Margin (Delta-method)	Std. Err.	z	P>|z|	[95% Conf.	Interval]
region						
1	**.9994512**	**.0000439**	**2.3e+04**	**0.000**	**.9993652**	**.9995372**
2	**.9996682**	**.0000317**	**3.2e+04**	**0.000**	**.999606**	**.9997303**

450 . margins region, post

Predictive margins Number of obs = **2100195**
Model VCE : **Linearized**

Expression : **Pr(uc_cases_new), predict()**

	Margin (Delta-method)	Std. Err.	z	P>|z|	[95% Conf.	Interval]
region						
1	**.9994512**	**.0000439**	**2.3e+04**	**0.000**	**.9993652**	**.9995372**
2	**.9996682**	**.0000317**	**3.2e+04**	**0.000**	**.999606**	**.9997303**

451 . // OVERALL INCIDENCE RATE UC only: North REGION
unrecognized command: / invalid command name
r(199);

452 . lincom 1-1.region

(1) **- 1bn.region = -1**

	Coef.	Std. Err.	z	P>|z|	[95% Conf.	Interval]
(1)	**.0005488**	**.0000439**	**12.50**	**0.000**	**.0004628**	**.0006348**

453 . // OVERALL INCIDENCE RATE UC only : South REGION
unrecognized command: / invalid command name
r(199);

454 . lincom 1-2.region

(1) **- 2.region = -1**

	Coef.	Std. Err.	z	P>|z|	[95% Conf.	Interval]
(1)	**.0003318**	**.0000317**	**10.47**	**0.000**	**.0002697**	**.000394**

```
455 .
456 . //Comparison within North Region between Winter and Summer Season (Reference: WInter month)
    unrecognized command:  / invalid command name
    r(199);

457 . svy: logistic uc_cases_new  r1s
    (running logistic on estimation sample)

    Survey: Logistic regression

    Number of strata   =        37                  Number of obs      =     980627
    Number of PSUs     =       273                  Population size    =  4877690.8
                                                    Design df          =        236
                                                    F(   1,    236)    =       1.99
                                                    Prob > F           =     0.1598

    ------------------------------------------------------------------------------
                 |             Linearized
    uc_cases_new | Odds Ratio   Std. Err.      t    P>|t|     [95% Conf. Interval]
    -------------+----------------------------------------------------------------
             r1s |   1.125632   .0944724     1.41   0.160     .9540871     1.32802
           _cons |   1527.088   211.3287    52.98   0.000     1162.684    2005.701
    ------------------------------------------------------------------------------
    Note: strata with single sampling unit centered at overall mean.

458 . //Comparison within South Region between Winter and Summer Season (Reference: WInter month)
    unrecognized command:  / invalid command name
    r(199);

459 . svy: logistic uc_cases_new  r2s
    (running logistic on estimation sample)

    Survey: Logistic regression

    Number of strata   =        35                  Number of obs      =    1119568
    Number of PSUs     =       334                  Population size    =  5411634.2
                                                    Design df          =        299
                                                    F(   1,    299)    =       0.02
                                                    Prob > F           =     0.8868

    ------------------------------------------------------------------------------
                 |             Linearized
    uc_cases_new | Odds Ratio   Std. Err.      t    P>|t|     [95% Conf. Interval]
    -------------+----------------------------------------------------------------
             r2s |   .9844932   .1079536    -0.14   0.887     .7934065    1.221602
           _cons |   3082.999   556.1097    44.54   0.000     2161.773      4396.8
    ------------------------------------------------------------------------------
    Note: strata with single sampling unit centered at overall mean.

460 .
461 . //*****************************************************************************************
    unrecognized command:  / invalid command name
```

```
r(199);

462 . // PART3. CD Cases only
unrecognized command:  / invalid command name
r(199);

463 . ****************************************************************************************

464 . svy: logistic cd_cases_new i.region season_new
(running logistic on estimation sample)

Survey: Logistic regression

Number of strata   =        59                 Number of obs      =    2100195
Number of PSUs     =       607                 Population size    =   10289325
                                               Design df          =        548
                                               F(   2,    547)    =      12.46
                                               Prob > F           =     0.0000
```

cd_cases_new	Odds Ratio	Linearized Std. Err.	t	P>|t|	[95% Conf.	Interval]
2.region	**1.588351**	**.1474104**	**4.99**	**0.000**	**1.323653**	**1.905983**
season_new	**1.004275**	**.0515766**	**0.08**	**0.934**	**.9079055**	**1.110873**
_cons	**927.6276**	**92.21741**	**68.73**	**0.000**	**763.0739**	**1127.667**

```
Note: strata with single sampling unit centered at overall mean.

465 . svy: logistic cd_cases_new i.region
(running logistic on estimation sample)

Survey: Logistic regression

Number of strata   =        59                 Number of obs      =    4819620
Number of PSUs     =       658                 Population size    =   23552265
                                               Design df          =        599
                                               F(   1,    599)    =      25.88
                                               Prob > F           =     0.0000
```

cd_cases_new	Odds Ratio	Linearized Std. Err.	t	P>|t|	[95% Conf.	Interval]
2.region	**1.499233**	**.1193489**	**5.09**	**0.000**	**1.282244**	**1.752942**
_cons	**950.8768**	**51.77611**	**125.94**	**0.000**	**854.4403**	**1058.198**

```
Note: strata with single sampling unit centered at overall mean.

466 . margins region

Adjusted predictions                             Number of obs   =    4819620
Model VCE    : Linearized
```

```
    Expression   : Pr(cd_cases_new), predict()
```

	Margin	Delta-method Std. Err.	z	P>|z|	[95% Conf.	Interval]
region						
1	**.9989494**	**.0000571**	**1.7e+04**	**0.000**	**.9988374**	**.9990614**
2	**.999299**	**.0000407**	**2.5e+04**	**0.000**	**.9992193**	**.9993788**

```
467 . margins region, post

    Adjusted predictions                              Number of obs   =    4819620
    Model VCE    : Linearized

    Expression   : Pr(cd_cases_new), predict()
```

	Margin	Delta-method Std. Err.	z	P>|z|	[95% Conf.	Interval]
region						
1	**.9989494**	**.0000571**	**1.7e+04**	**0.000**	**.9988374**	**.9990614**
2	**.999299**	**.0000407**	**2.5e+04**	**0.000**	**.9992193**	**.9993788**

```
468 . // OVERALL INCIDENCE RATE CD only: North REGION
    unrecognized command:  / invalid command name
    r(199);

469 . lincom 1-1.region

     ( 1)  - 1bn.region = -1
```

	Coef.	Std. Err.	z	P>|z|	[95% Conf.	Interval]
(1)	**.0010506**	**.0000571**	**18.38**	**0.000**	**.0009386**	**.0011626**

```
470 . // OVERALL INCIDENCE RATE CD only: South REGION
    unrecognized command:  / invalid command name
    r(199);

471 . lincom 1-2.region

     ( 1)  - 2.region = -1
```

	Coef.	Std. Err.	z	P>|z|	[95% Conf.	Interval]
(1)	**.000701**	**.0000407**	**17.22**	**0.000**	**.0006212**	**.0007807**

```
472 .
473 . //Comparison within North Region between Winter and Summer Season (Reference: WInter month)
    unrecognized command:  / invalid command name
    r(199);

474 . svy: logistic cd_cases_new  r1s
    (running logistic on estimation sample)

    Survey: Logistic regression

    Number of strata   =         37                 Number of obs      =     980627
    Number of PSUs     =        273                 Population size    =  4877690.8
                                                    Design df          =        236
                                                    F(   1,    236)    =       0.55
                                                    Prob > F           =     0.4607

    ------------------------------------------------------------------------------
                 |             Linearized
    cd_cases_new | Odds Ratio   Std. Err.      t    P>|t|     [95% Conf. Interval]
    -------------+----------------------------------------------------------------
             r1s |   1.053654   .0745223     0.74   0.461     .9166098    1.211189
           _cons |   863.2286    106.551    54.77   0.000     676.8895    1100.865
    ------------------------------------------------------------------------------
    Note: strata with single sampling unit centered at overall mean.

475 . //Comparison within South Region between Winter and Summer Season (Reference: WInter month)
    unrecognized command:  / invalid command name
    r(199);

476 . svy: logistic cd_cases_new  r2s
    (running logistic on estimation sample)

    Survey: Logistic regression

    Number of strata   =         35                 Number of obs      =    1119568
    Number of PSUs     =        334                 Population size    =  5411634.2
                                                    Design df          =        299
                                                    F(   1,    299)    =       0.85
                                                    Prob > F           =     0.3580

    ------------------------------------------------------------------------------
                 |             Linearized
    cd_cases_new | Odds Ratio   Std. Err.      t    P>|t|     [95% Conf. Interval]
    -------------+----------------------------------------------------------------
             r2s |   .9376287   .0655864    -0.92   0.358     .8170488    1.076004
           _cons |   1633.699   221.3345    54.61   0.000     1251.359    2132.858
    ------------------------------------------------------------------------------
    Note: strata with single sampling unit centered at overall mean.

477 . clear

478 . //YEAR 2006
    unrecognized command:  / invalid command name
```

```
    r(199);

479 . use "U:\RKunnavakkam\Adam_Stein\2006\UC_CD_2006.req.dta", clear

480 . //**********************************************************************
    unrecognized command:  / invalid command name
    r(199);

481 .
482 . // PART1. UC or CD cases
    unrecognized command:  / invalid command name
    r(199);

483 .
484 . //**********************************************************************
    unrecognized command:  / invalid command name
    r(199);

485 .
486 .
487 .
488 . svyset hospid [pweight=discwt], strata (nis_stratum) singleunit(centered)

          pweight: discwt
              VCE: linearized
      Single unit: centered
         Strata 1: nis_stratum
             SU 1: hospid
            FPC 1: <zero>

489 .
490 . svy: logistic uc_cd_new region season_new
    (running logistic on estimation sample)

    Survey: Logistic regression

    Number of strata   =        57                  Number of obs      =   2038691
    Number of PSUs     =       576                  Population size    =  10067234
                                                    Design df          =       519
                                                    F(   2,    518)    =     18.31
                                                    Prob > F           =    0.0000

    ------------------------------------------------------------------------------
                 |             Linearized
       uc_cd_new | Odds Ratio   Std. Err.      t    P>|t|     [95% Conf. Interval]
    -------------+----------------------------------------------------------------
          region |   1.472575   .0948534     6.01   0.000      1.29754    1.671223
      season_new |   .9882785   .0270012    -0.43   0.666     .9366318    1.042773
           _cons |   196.9481    20.2538    51.37   0.000     160.9204    241.0419
    ------------------------------------------------------------------------------
    Note: strata with single sampling unit centered at overall mean.

491 .
492 . svy: logistic uc_cd_new i.region
```

```
(running logistic on estimation sample)

Survey: Logistic regression

Number of strata   =        57                  Number of obs      =    4762963
Number of PSUs     =       628                  Population size    =   23289253
                                                Design df          =        571
                                                F(   1,    571)    =      26.67
                                                Prob > F           =     0.0000
```

uc_cd_new	Odds Ratio	Linearized Std. Err.	t	P>|t|	[95% Conf.	Interval]
2.region	1.370952	.0837628	5.16	0.000	1.215919	1.545751
_cons	294.0673	14.20497	117.66	0.000	267.4496	323.3341

Note: strata with single sampling unit centered at overall mean.

```
493 .
494 . margins region

Adjusted predictions                              Number of obs   =    4762963
Model VCE    : Linearized

Expression   : Pr(uc_cd_new), predict()
```

	Margin	Delta-method Std. Err.	z	P>|z|	[95% Conf.	Interval]
region						
1	.9966109	.0001632	6108.40	0.000	.9962912	.9969307
2	.9975257	.0000946	1.1e+04	0.000	.9973404	.997711

```
495 .
496 . margins region, post

Adjusted predictions                              Number of obs   =    4762963
Model VCE    : Linearized

Expression   : Pr(uc_cd_new), predict()
```

	Margin	Delta-method Std. Err.	z	P>|z|	[95% Conf.	Interval]
region						
1	.9966109	.0001632	6108.40	0.000	.9962912	.9969307
2	.9975257	.0000946	1.1e+04	0.000	.9973404	.997711

```
497 .
```

```
498 . // OVERALL INCIDENCE RATE UC or CD cases : North REGION
    unrecognized command:  / invalid command name
    r(199);

499 .
500 . lincom 1-1.region

     ( 1)  - 1bn.region = -1
```

	Coef.	Std. Err.	z	P>|z|	[95% Conf. Interval]	
(1)	**.0033891**	**.0001632**	**20.77**	**0.000**	**.0030693**	**.0037088**

```
501 .
502 . // OVERALL INCIDENCE RATE UC or CD cases : South REGION
    unrecognized command:  / invalid command name
    r(199);

503 .
504 . lincom 1-2.region

     ( 1)  - 2.region = -1
```

	Coef.	Std. Err.	z	P>|z|	[95% Conf. Interval]	
(1)	**.0024743**	**.0000946**	**26.17**	**0.000**	**.002289**	**.0026596**

```
505 .
506 . // This gives results for within region comparing season1 and season 2
    unrecognized command:  / invalid command name
    r(199);

507 .
508 . //Comparison within North Region between Winter and Summer Season (Reference: WInter month)
    unrecognized command:  / invalid command name
    r(199);

509 .
510 . svy: logistic uc_cd_new  r1s
    (running logistic on estimation sample)

    Survey: Logistic regression

    Number of strata   =        36                 Number of obs      =    979030
    Number of PSUs     =       266                 Population size    = 5080610.2
                                                   Design df          =       230
                                                   F(   1,    230)    =      0.07
                                                   Prob > F           =    0.7899
```

uc_cd_new	Odds Ratio	Linearized Std. Err.	t	P>|t|	[95% Conf.	Interval]
r1s	**.9902392**	**.0364117**	**-0.27**	**0.790**	**.9210332**	**1.064645**
_cons	**289.1546**	**17.70983**	**92.53**	**0.000**	**256.2836**	**326.2416**

Note: strata with single sampling unit centered at overall mean.

```
511 .
512 . //Comparison within South Region between Winter and Summer Season (Reference: WInter month
    unrecognized command:  / invalid command name
    r(199);

513 .
514 . svy: logistic uc_cd_new   r2s
    (running logistic on estimation sample)

    Survey: Logistic regression

    Number of strata   =        34                  Number of obs      =   1059661
    Number of PSUs     =       310                  Population size    = 4986623.8
                                                    Design df          =       276
                                                    F(   1,    276)    =      0.14
                                                    Prob > F           =    0.7111
```

uc_cd_new	Odds Ratio	Linearized Std. Err.	t	P>|t|	[95% Conf.	Interval]
r2s	**.9853515**	**.0392231**	**-0.37**	**0.711**	**.9110849**	**1.065672**
_cons	**428.9823**	**32.85699**	**79.14**	**0.000**	**368.9404**	**498.7954**

Note: strata with single sampling unit centered at overall mean.

```
515 .
516 .
517 .
518 . //**********************************************************************************************
    unrecognized command:  / invalid command name
    r(199);

519 .
520 . // PART2. UC cases only
    unrecognized command:  / invalid command name
    r(199);

521 .
522 . //*********************************************************************************************
    unrecognized command:  / invalid command name
    r(199);

523 .
524 . svy: logistic uc_cases_new i.region season_new
    (running logistic on estimation sample)
```

```
Survey: Logistic regression

Number of strata   =        57                  Number of obs      =   2038691
Number of PSUs     =       576                  Population size    =  10067234
                                                Design df          =       519
                                                F(   2,    518)    =     16.37
                                                Prob > F           =    0.0000
```

uc_cases_new	Odds Ratio	Linearized Std. Err.	t	P>|t|	[95% Conf.	Interval]
2.region	1.516607	.1103929	5.72	0.000	1.314528	1.749751
season_new	.9714783	.0426078	-0.66	0.510	.8912782	1.058895
_cons	751.2585	59.55004	83.54	0.000	642.9237	877.848

Note: strata with single sampling unit centered at overall mean.

```
525 .
526 . margins region

Predictive margins                                Number of obs   =    2038691
Model VCE     : Linearized

Expression    : Pr(uc_cases_new), predict()
```

	Margin	Delta-method Std. Err.	z	P>|z|	[95% Conf.	Interval]
region						
1	.9986116	.0000693	1.4e+04	0.000	.9984757	.9987475
2	.9990841	.0000506	2.0e+04	0.000	.998985	.9991832

```
527 .
528 . margins region, post

Predictive margins                                Number of obs   =    2038691
Model VCE     : Linearized

Expression    : Pr(uc_cases_new), predict()
```

	Margin	Delta-method Std. Err.	z	P>|z|	[95% Conf.	Interval]
region						
1	.9986116	.0000693	1.4e+04	0.000	.9984757	.9987475
2	.9990841	.0000506	2.0e+04	0.000	.998985	.9991832

```
529 .
```

```
530 . // OVERALL INCIDENCE RATE UC only: North REGION
    unrecognized command:  / invalid command name
    r(199);

531 .
532 . lincom 1-1.region

     ( 1)  - 1bn.region = -1
```

	Coef.	Std. Err.	z	P>|z|	[95% Conf.	Interval]
(1)	**.0013884**	**.0000693**	**20.02**	**0.000**	**.0012525**	**.0015243**

```
533 .
534 . // OVERALL INCIDENCE RATE UC only : South REGION
    unrecognized command:  / invalid command name
    r(199);

535 .
536 . lincom 1-2.region

     ( 1)  - 2.region = -1
```

	Coef.	Std. Err.	z	P>|z|	[95% Conf.	Interval]
(1)	**.0009159**	**.0000506**	**18.11**	**0.000**	**.0008168**	**.001015**

```
537 .
538 .
539 .
540 . //Comparison within North Region between Winter and Summer Season (Reference: WInter month)
    unrecognized command:  / invalid command name
    r(199);

541 .
542 . svy: logistic uc_cases_new  r1s
    (running logistic on estimation sample)

    Survey: Logistic regression

    Number of strata   =        36                Number of obs      =    979030
    Number of PSUs     =       266                Population size    = 5080610.2
                                                  Design df          =       230
                                                  F(   1,    230)    =      0.26
                                                  Prob > F           =    0.6134
```

uc_cases_new	Odds Ratio	Linearized Std. Err.	t	P>|t|	[95% Conf.	Interval]

```
         r1s |   .9727597   .0531043    -0.51   0.613     .8735575    1.083227
       _cons |    749.761    66.7751    74.33   0.000     629.0892      893.58
------------------------------------------------------------------------------
Note: strata with single sampling unit centered at overall mean.

543 .
544 . //Comparison within South Region between Winter and Summer Season (Reference: WInter month)
unrecognized command:  / invalid command name
r(199);

545 .
546 . svy: logistic uc_cases_new  r2s
(running logistic on estimation sample)

Survey: Logistic regression

Number of strata   =        34                  Number of obs      =   1059661
Number of PSUs     =       310                  Population size    = 4986623.8
                                                Design df          =       276
                                                F(   1,    276)    =      0.19
                                                Prob > F           =    0.6645

------------------------------------------------------------------------------
             |             Linearized
uc_cases_new | Odds Ratio   Std. Err.      t    P>|t|     [95% Conf. Interval]
-------------+----------------------------------------------------------------
         r2s |   .9695043    .069159    -0.43   0.665     .8424854    1.115673
       _cons |   1142.853   138.7405    58.00   0.000     899.9134    1451.376
------------------------------------------------------------------------------
Note: strata with single sampling unit centered at overall mean.

547 .
548 .
549 .
550 . //*****************************************************************************************
unrecognized command:  / invalid command name
r(199);

551 .
552 . // PART3. CD Cases only
unrecognized command:  / invalid command name
r(199);

553 .
554 . *********************************************************************************************

555 .
556 . svy: logistic cd_cases_new i.region season_new
(running logistic on estimation sample)

Survey: Logistic regression

Number of strata   =        57                  Number of obs      =   2038691
Number of PSUs     =       576                  Population size    =  10067234
```

```
                                                 Design df          =        519
                                                 F(   2,    518)    =      13.63
                                                 Prob > F           =     0.0000
```

cd_cases_new	Odds Ratio	Linearized Std. Err.	t	P>|t|	[95% Conf.	Interval]
2.region	1.452103	.1058831	5.12	0.000	1.258303	1.675752
season_new	1.012645	.0346213	0.37	0.713	.9468633	1.082996
_cons	435.1751	28.9385	91.37	0.000	381.881	495.9067

Note: strata with single sampling unit centered at overall mean.

```
557 .
558 . svy: logistic cd_cases_new i.region
    (running logistic on estimation sample)

    Survey: Logistic regression

    Number of strata   =        57              Number of obs      =    4762963
    Number of PSUs     =       628              Population size    =   23289253
                                                Design df          =        571
                                                F(   1,    571)    =      22.26
                                                Prob > F           =     0.0000
```

cd_cases_new	Odds Ratio	Linearized Std. Err.	t	P>|t|	[95% Conf.	Interval]
2.region	1.381998	.0947653	4.72	0.000	1.207856	1.581245
_cons	456.0802	24.60863	113.47	0.000	410.2188	507.0688

Note: strata with single sampling unit centered at overall mean.

```
559 .
560 . margins region

    Adjusted predictions                           Number of obs   =    4762963
    Model VCE    : Linearized

    Expression   : Pr(cd_cases_new), predict()
```

	Margin	Delta-method Std. Err.	z	P>|z|	[95% Conf.	Interval]
region						
1	.9978122	.0001178	8471.22	0.000	.9975813	.9980431
2	.998416	.0000674	1.5e+04	0.000	.9982838	.9985481

```
561 .
562 . margins region, post
```

```
Adjusted predictions                              Number of obs   =    4762963
Model VCE    : Linearized

Expression   : Pr(cd_cases_new), predict()
```

	Delta-method Margin	Std. Err.	z	P>|z|	[95% Conf.	Interval]
region						
1	.9978122	.0001178	8471.22	0.000	.9975813	.9980431
2	.998416	.0000674	1.5e+04	0.000	.9982838	.9985481

```
563 .
564 . // OVERALL INCIDENCE RATE CD only: North REGION
    unrecognized command:  / invalid command name
    r(199);

565 .
566 . lincom 1-1.region

     ( 1)  - 1bn.region = -1
```

	Coef.	Std. Err.	z	P>|z|	[95% Conf.	Interval]
(1)	.0021878	.0001178	18.57	0.000	.0019569	.0024187

```
567 .
568 . // OVERALL INCIDENCE RATE CD only: South REGION
    unrecognized command:  / invalid command name
    r(199);

569 .
570 . lincom 1-2.region

     ( 1)  - 2.region = -1
```

	Coef.	Std. Err.	z	P>|z|	[95% Conf.	Interval]
(1)	.001584	.0000674	23.49	0.000	.0014519	.0017162

```
571 .
572 .
573 .
574 . //Comparison within North Region between Winter and Summer Season (Reference: WInter month)
    unrecognized command:  / invalid command name
    r(199);
```

```
575 .
576 . svy: logistic cd_cases_new  r1s
    (running logistic on estimation sample)

    Survey: Logistic regression

    Number of strata   =        36                  Number of obs      =    979030
    Number of PSUs     =       266                  Population size    = 5080610.2
                                                    Design df          =       230
                                                    F(   1,    230)    =      0.14
                                                    Prob > F           =    0.7049

    ------------------------------------------------------------------------------
                 |             Linearized
    cd_cases_new | Odds Ratio   Std. Err.      t    P>|t|     [95% Conf. Interval]
    -------------+----------------------------------------------------------------
             r1s |   1.016446    .043725     0.38   0.705     .9338436    1.106355
           _cons |   432.7309   31.10434    84.45   0.000      375.587    498.5689
    ------------------------------------------------------------------------------
    Note: strata with single sampling unit centered at overall mean.

577 .
578 . //Comparison within South Region between Winter and Summer Season (Reference: WInter month)
    unrecognized command:  / invalid command name
    r(199);

579 .
580 . svy: logistic cd_cases_new  r2s
    (running logistic on estimation sample)

    Survey: Logistic regression

    Number of strata   =        34                  Number of obs      =   1059661
    Number of PSUs     =       310                  Population size    = 4986623.8
                                                    Design df          =       276
                                                    F(   1,    276)    =      0.02
                                                    Prob > F           =    0.8985

    ------------------------------------------------------------------------------
                 |             Linearized
    cd_cases_new | Odds Ratio   Std. Err.      t    P>|t|     [95% Conf. Interval]
    -------------+----------------------------------------------------------------
             r2s |   1.007054    .055459     0.13   0.899     .9035875    1.122368
           _cons |   637.1666   60.93132    67.52   0.000     527.8315    769.1493
    ------------------------------------------------------------------------------
    Note: strata with single sampling unit centered at overall mean.

581 . clear

582 . use "U:\RKunnavakkam\Adam_Stein\2007\UC_CD_2007_req.dta", clear

583 . //*********************************************************************
    unrecognized command:  / invalid command name
    r(199);
```

```
584 .
585 . // PART1. UC or CD cases
    unrecognized command:  / invalid command name
    r(199);

586 .
587 . //*********************************************************************
    unrecognized command:  / invalid command name
    r(199);

588 .
589 .
590 .
591 . svyset hospid [pweight=discwt], strata (nis_stratum) singleunit(centered)

          pweight: discwt
              VCE: linearized
      Single unit: centered
         Strata 1: nis_stratum
             SU 1: hospid
            FPC 1: <zero>

592 .
593 . svy: logistic uc_cd_new region season_new
    (running logistic on estimation sample)

    Survey: Logistic regression

    Number of strata   =        58                  Number of obs      =   1933430
    Number of PSUs     =       580                  Population size    = 9684125.2
                                                    Design df          =       522
                                                    F(   2,    521)    =      4.97
                                                    Prob > F           =    0.0073

    ------------------------------------------------------------------------------
                 |             Linearized
       uc_cd_new | Odds Ratio   Std. Err.      t    P>|t|     [95% Conf. Interval]
    -------------+----------------------------------------------------------------
          region |   1.326665   .1188729     3.15   0.002     1.112535    1.582007
      season_new |   1.013913   .0297604     0.47   0.638     .9571023    1.074097
           _cons |   208.7115   26.99503    41.29   0.000     161.8807    269.0902
    ------------------------------------------------------------------------------
    Note: strata with single sampling unit centered at overall mean.

594 .
595 . svy: logistic uc_cd_new i.region
    (running logistic on estimation sample)

    Survey: Logistic regression

    Number of strata   =        58                  Number of obs      =   4615779
    Number of PSUs     =       633                  Population size    =  23033762
                                                    Design df          =       575
```

```
                                          F(   1,    575)    =      7.48
                                          Prob > F           =    0.0064
```

uc_cd_new	Odds Ratio	Linearized Std. Err.	t	P>|t|	[95% Conf.	Interval]
2.region	**1.203646**	**.0815599**	**2.74**	**0.006**	**1.053657**	**1.374987**
_cons	**277.0198**	**11.57988**	**134.54**	**0.000**	**255.1844**	**300.7236**

Note: strata with single sampling unit centered at overall mean.

```
596 .
597 . margins region
```

Adjusted predictions   Number of obs   =   **4615779**
Model VCE   : **Linearized**

Expression   : **Pr(uc_cd_new), predict()**

	Margin	Delta-method Std. Err.	z	P>|z|	[95% Conf.	Interval]
region						
1	**.9964031**	**.0001498**	**6650.93**	**0.000**	**.9961095**	**.9966968**
2	**.9970099**	**.0001594**	**6253.14**	**0.000**	**.9966974**	**.9973224**

```
598 .
599 . margins region, post
```

Adjusted predictions   Number of obs   =   **4615779**
Model VCE   : **Linearized**

Expression   : **Pr(uc_cd_new), predict()**

	Margin	Delta-method Std. Err.	z	P>|z|	[95% Conf.	Interval]
region						
1	**.9964031**	**.0001498**	**6650.93**	**0.000**	**.9961095**	**.9966968**
2	**.9970099**	**.0001594**	**6253.14**	**0.000**	**.9966974**	**.9973224**

```
600 .
601 . // OVERALL INCIDENCE RATE UC or CD cases : North REGION
```

unrecognized command: / invalid command name
r(199);

```
602 .
603 . lincom 1-1.region
```

```
( 1)  - 1bn.region = -1
```

	Coef.	Std. Err.	z	P>|z|	[95% Conf.	Interval]
(1)	**.0035969**	**.0001498**	**24.01**	**0.000**	**.0033032**	**.0038905**

```
604 .
605 . // OVERALL INCIDENCE RATE UC or CD cases : South REGION
    unrecognized command:  / invalid command name
    r(199);

606 .
607 . lincom 1-2.region

 ( 1)  - 2.region = -1
```

	Coef.	Std. Err.	z	P>|z|	[95% Conf.	Interval]
(1)	**.0029901**	**.0001594**	**18.75**	**0.000**	**.0026776**	**.0033026**

```
608 .
609 . // This gives results for within region comparing season1 and season 2
    unrecognized command:  / invalid command name
    r(199);

610 .
611 . //Comparison within North Region between Winter and Summer Season (Reference: WInter month)
    unrecognized command:  / invalid command name
    r(199);

612 .
613 . svy: logistic uc_cd_new  r1s
    (running logistic on estimation sample)

    Survey: Logistic regression

    Number of strata   =        36                Number of obs      =    947122
    Number of PSUs     =       273                Population size    = 4953418.2
                                                  Design df          =       237
                                                  F(   1,    237)    =      0.01
                                                  Prob > F           =    0.9414
```

uc_cd_new	Odds Ratio	Linearized Std. Err.	t	P>|t|	[95% Conf.	Interval]
r1s	**1.002725**	**.0371051**	**0.07**	**0.941**	**.9322277**	**1.078553**
_cons	**281.5517**	**18.24763**	**87.03**	**0.000**	**247.8037**	**319.8957**

Note: strata with single sampling unit centered at overall mean.

```
614 .
615 . //Comparison within South Region between Winter and Summer Season (Reference: WInter month
    unrecognized command:  / invalid command name
    r(199);

616 .
617 . svy: logistic uc_cd_new   r2s
    (running logistic on estimation sample)

    Survey: Logistic regression

    Number of strata   =        33                  Number of obs      =    986308
    Number of PSUs     =       307                  Population size    =   4730707
                                                    Design df          =       274
                                                    F(   1,    274)    =      0.37
                                                    Prob > F           =    0.5439

    ------------------------------------------------------------------------------
                 |             Linearized
       uc_cd_new | Odds Ratio   Std. Err.      t    P>|t|     [95% Conf. Interval]
    -------------+----------------------------------------------------------------
             r2s |   1.029635    .049478     0.61   0.544     .9366948    1.131796
           _cons |    358.985   28.40643    74.35   0.000     307.2006    419.4987
    ------------------------------------------------------------------------------
    Note: strata with single sampling unit centered at overall mean.

618 .
619 .
620 .
621 . //****************************************************************************************
    unrecognized command:  / invalid command name
    r(199);

622 .
623 . // PART2. UC cases only
    unrecognized command:  / invalid command name
    r(199);

624 .
625 . //***************************************************************************************
    unrecognized command:  / invalid command name
    r(199);

626 .
627 . svy: logistic uc_cases_new i.region season_new
    (running logistic on estimation sample)

    Survey: Logistic regression

    Number of strata   =        58                  Number of obs      =   1933430
    Number of PSUs     =       580                  Population size    = 9684125.2
                                                    Design df          =       522
                                                    F(   2,    521)    =      4.11
```

```
                                              Prob > F          =    0.0170

------------------------------------------------------------------------------
             |             Linearized
uc_cases_new | Odds Ratio   Std. Err.      t    P>|t|     [95% Conf. Interval]
-------------+----------------------------------------------------------------
    2.region |   1.311948   .1242507     2.87   0.004     1.089217    1.580224
  season_new |   1.009016   .0426247     0.21   0.832     .9286594    1.096326
       _cons |   677.9933   49.49996    89.29   0.000     587.4016    782.5564
------------------------------------------------------------------------------
Note: strata with single sampling unit centered at overall mean.

628 .
629 . margins region

Predictive margins                                Number of obs     =    1933430
Model VCE    : Linearized

Expression   : Pr(uc_cases_new), predict()

------------------------------------------------------------------------------
             |            Delta-method
             |     Margin   Std. Err.      z    P>|z|     [95% Conf. Interval]
-------------+----------------------------------------------------------------
      region |
          1  |   .9985469   .0000613  1.6e+04   0.000     .9984268    .9986671
          2  |   .9988921   .0000937  1.1e+04   0.000     .9987084    .9990757
------------------------------------------------------------------------------

630 .
631 . margins region, post

Predictive margins                                Number of obs     =    1933430
Model VCE    : Linearized

Expression   : Pr(uc_cases_new), predict()

------------------------------------------------------------------------------
             |            Delta-method
             |     Margin   Std. Err.      z    P>|z|     [95% Conf. Interval]
-------------+----------------------------------------------------------------
      region |
          1  |   .9985469   .0000613  1.6e+04   0.000     .9984268    .9986671
          2  |   .9988921   .0000937  1.1e+04   0.000     .9987084    .9990757
------------------------------------------------------------------------------

632 .
633 . // OVERALL INCIDENCE RATE UC only: North REGION
unrecognized command:  / invalid command name
r(199);

634 .
635 . lincom 1-1.region
```

(1) **- 1bn.region = -1**

	Coef.	Std. Err.	z	P>|z|	[95% Conf. Interval]	
(1)	**.0014531**	**.0000613**	**23.71**	**0.000**	**.0013329**	**.0015732**

636 .
637 . // OVERALL INCIDENCE RATE UC only : South REGION
unrecognized command: / invalid command name
r(199);

638 .
639 . lincom 1-2.region

(1) **- 2.region = -1**

	Coef.	Std. Err.	z	P>|z|	[95% Conf. Interval]	
(1)	**.0011079**	**.0000937**	**11.82**	**0.000**	**.0009243**	**.0012916**

640 .
641 .
642 .
643 . //Comparison within North Region between Winter and Summer Season (Reference: WInter month)
unrecognized command: / invalid command name
r(199);

644 .
645 . svy: logistic uc_cases_new r1s
(running logistic on estimation sample)

Survey: Logistic regression

Number of strata =	**36**	Number of obs =	**947122**
Number of PSUs =	**273**	Population size =	**4953418.2**
		Design df =	**237**
		F(**1**, **237**) =	**0.00**
		Prob > F =	**0.9601**

uc_cases_new	Odds Ratio	Linearized Std. Err.	t	P>|t|	[95% Conf. Interval]	
r1s	**.9971511**	**.056741**	**-0.05**	**0.960**	**.8914076**	**1.115438**
_cons	**690.1775**	**63.55902**	**70.98**	**0.000**	**575.666**	**827.4676**

Note: strata with single sampling unit centered at overall mean.

646 .
647 . //Comparison within South Region between Winter and Summer Season (Reference: WInter month)

```
unrecognized command:  / invalid command name
r(199);

648 .
649 . svy: logistic uc_cases_new  r2s
(running logistic on estimation sample)

Survey: Logistic regression

Number of strata   =        33                 Number of obs      =    986308
Number of PSUs     =       307                 Population size    =   4730707
                                               Design df          =       274
                                               F(   1,    274)    =      0.16
                                               Prob > F           =    0.6904
```

uc_cases_new	Odds Ratio	Linearized Std. Err.	t	P>|t|	[95% Conf.	Interval]
r2s	1.025532	.0648384	0.40	0.690	.9055111	1.16146
_cons	868.1414	102.1569	57.50	0.000	688.6247	1094.456

```
Note: strata with single sampling unit centered at overall mean.

650 .
651 .
652 .
653 . //*************************************************************************************
unrecognized command:  / invalid command name
r(199);

654 .
655 . // PART3. CD Cases only
unrecognized command:  / invalid command name
r(199);

656 .
657 . ********************************************************************************************

658 .
659 . svy: logistic cd_cases_new i.region season_new
(running logistic on estimation sample)

Survey: Logistic regression

Number of strata   =        58                 Number of obs      =   1933430
Number of PSUs     =       580                 Population size    = 9684125.2
                                               Design df          =       522
                                               F(   2,    521)    =      5.11
                                               Prob > F           =    0.0063
```

cd_cases_new	Odds Ratio	Linearized Std. Err.	t	P>|t|	[95% Conf.	Interval]

2.region	**1.358395**	**.1300134**	**3.20**	**0.001**	**1.125557**	**1.639399**
season_new	**1.026024**	**.0381333**	**0.69**	**0.490**	**.9537795**	**1.10374**
_cons	**424.771**	**30.84514**	**83.34**	**0.000**	**368.2989**	**489.9021**

Note: strata with single sampling unit centered at overall mean.

```
660 .
661 . svy: logistic cd_cases_new i.region
(running logistic on estimation sample)

Survey: Logistic regression

Number of strata   =        58                  Number of obs      =   4615779
Number of PSUs     =       633                  Population size    =  23033762
                                                Design df          =       575
                                                F(   1,    575)    =      6.67
                                                Prob > F           =    0.0101
```

cd_cases_new	Odds Ratio	Linearized Std. Err.	t	P>|t|	[95% Conf.	Interval]
2.region	**1.205752**	**.0873841**	**2.58**	**0.010**	**1.045777**	**1.390199**
_cons	**429.3559**	**20.80226**	**125.12**	**0.000**	**390.382**	**472.2208**

Note: strata with single sampling unit centered at overall mean.

```
662 .
663 . margins region

Adjusted predictions                            Number of obs     =    4615779
Model VCE    : Linearized

Expression   : Pr(cd_cases_new), predict()
```

	Margin	Delta-method Std. Err.	z	P>|z|	[95% Conf.	Interval]
region						
1	**.9976763**	**.0001123**	**8882.49**	**0.000**	**.9974562**	**.9978965**
2	**.9980721**	**.0001039**	**9607.73**	**0.000**	**.9978685**	**.9982757**

```
664 .
665 . margins region, post

Adjusted predictions                            Number of obs     =    4615779
Model VCE    : Linearized

Expression   : Pr(cd_cases_new), predict()
```

	Delta-method Margin	Std. Err.	z	P>|z|	[95% Conf. Interval]	
region						
1	**.9976763**	**.0001123**	**8882.49**	**0.000**	**.9974562**	**.9978965**
2	**.9980721**	**.0001039**	**9607.73**	**0.000**	**.9978685**	**.9982757**

666 .

667 . // OVERALL INCIDENCE RATE CD only: North REGION

unrecognized command: / invalid command name

r(199);

668 .

669 . lincom 1-1.region

(1) **- 1bn.region = -1**

	Coef.	Std. Err.	z	P>|z|	[95% Conf. Interval]	
(1)	**.0023237**	**.0001123**	**20.69**	**0.000**	**.0021035**	**.0025438**

670 .

671 . // OVERALL INCIDENCE RATE CD only: South REGION

unrecognized command: / invalid command name

r(199);

672 .

673 . lincom 1-2.region

(1) **- 2.region = -1**

	Coef.	Std. Err.	z	P>|z|	[95% Conf. Interval]	
(1)	**.0019279**	**.0001039**	**18.56**	**0.000**	**.0017243**	**.0021315**

674 .

675 .

676 .

677 . //Comparison within North Region between Winter and Summer Season (Reference: WInter month)

unrecognized command: / invalid command name

r(199);

678 .

679 . svy: logistic cd_cases_new r1s

(running logistic on estimation sample)

Survey: Logistic regression

Number of strata = **36** Number of obs = **947122**

```
Number of PSUs     =       273                  Population size   = 4953418.2
                                                Design df         =       237
                                                F(   1,    237)   =      0.13
                                                Prob > F          =    0.7160
```

cd_cases_new	Odds Ratio	Linearized Std. Err.	t	P>|t|	[95% Conf.	Interval]
r1s	1.016294	.0450989	0.36	0.716	.9312212	1.109139
_cons	430.893	34.25585	76.30	0.000	368.4273	503.9495

```
Note: strata with single sampling unit centered at overall mean.

680 .
681 . //Comparison within South Region between Winter and Summer Season (Reference: WInter month)
    unrecognized command:  / invalid command name
    r(199);

682 .
683 . svy: logistic cd_cases_new  r2s
    (running logistic on estimation sample)

    Survey: Logistic regression

    Number of strata   =        33                  Number of obs     =    986308
    Number of PSUs     =       307                  Population size   =   4730707
                                                    Design df         =       274
                                                    F(   1,    274)   =      0.37
                                                    Prob > F          =    0.5432
```

cd_cases_new	Odds Ratio	Linearized Std. Err.	t	P>|t|	[95% Conf.	Interval]
r2s	1.040006	.0670152	0.61	0.543	.9161015	1.18067
_cons	565.4657	52.89364	67.75	0.000	470.3614	679.7996

```
Note: strata with single sampling unit centered at overall mean.

684 . log  close
          name:  <unnamed>
           log:  U:\RKunnavakkam\Adam_Stein\output.smcl
      log type:  smcl
     closed on:  20 Feb 2013, 11:45:25
```
